# Neuropathophysiological Mechanisms and Treatment Strategies for Post-traumatic Epilepsy

**DOI:** 10.3389/fnmol.2021.612073

**Published:** 2021-02-23

**Authors:** Shaunik Sharma, Grant Tiarks, Joseph Haight, Alexander G. Bassuk

**Affiliations:** Medical Laboratories, Department of Pediatrics, University of Iowa, Iowa City, IA, United States

**Keywords:** traumatic brain injury, post-traumatic epilepsy, excitotoxicity, neuroinflammation, oxidative stress, neurodegeneration, immune response, clinical management

## Abstract

Traumatic brain injury (TBI) is a leading cause of death in young adults and a risk factor for acquired epilepsy. Severe TBI, after a period of time, causes numerous neuropsychiatric and neurodegenerative problems with varying comorbidities; and brain homeostasis may never be restored. As a consequence of disrupted equilibrium, neuropathological changes such as circuit remodeling, reorganization of neural networks, changes in structural and functional plasticity, predisposition to synchronized activity, and post-translational modification of synaptic proteins may begin to dominate the brain. These pathological changes, over the course of time, contribute to conditions like Alzheimer disease, dementia, anxiety disorders, and post-traumatic epilepsy (PTE). PTE is one of the most common, devastating complications of TBI; and of those affected by a severe TBI, more than 50% develop PTE. The etiopathology and mechanisms of PTE are either unknown or poorly understood, which makes treatment challenging. Although anti-epileptic drugs (AEDs) are used as preventive strategies to manage TBI, control acute seizures and prevent development of PTE, their efficacy in PTE remains controversial. In this review, we discuss novel mechanisms and risk factors underlying PTE. We also discuss dysfunctions of neurovascular unit, cell-specific neuroinflammatory mediators and immune response factors that are vital for epileptogenesis after TBI. Finally, we describe current and novel treatments and management strategies for preventing PTE.

## Introduction

More than 3 million people in United States suffer a TBI each year. Among these cases, 80% are mild, 10% moderate, and about 10% severe, accounting for ∼300,000 hospitalizations and ∼50,000 fatalities, annually (Maas et al., 2017). Many traumatic brain injuries cause long-term disabilities, cognitive decline, psychiatric illness, and post-traumatic disorders. About 35% of TBI result from falls, 17% from motor vehicle accidents, and 10% from assaults, while in 21% of the cases, the cause was not recorded (Ding et al., 2016; Centers for Disease Control and Prevention, 2019). Incidence rates are higher in both males and females up to 9 years of age, during teen years, and towards the end of life (>74 years of age). Approximately, 2% of U.S. population live with long-lasting disabilities stemming from TBI; and is one of the single greatest causes of deaths and permanent disability in people under the age of 45 (Maas et al., 2017). The total estimated annual cost for TBI treatment is over $56.3 billion (Faul and Coronado, 2015; Maas et al., 2017). Currently, no available therapies can limit secondary injury or foster repair and regeneration.

Traumatic brain injury can trigger seizures and account for 4% of epilepsy cases (Gupta et al., 2014). New-onset symptomatic epilepsy in adolescents and young adults is most often caused by developmental disorders, infections, skull fracture, intracranial hemorrhage, and subarachnoid or subdural hemorrhage. In contrast, amongst older populations, intracranial hematoma, strokes and tumors are more common causes (Mahler et al., 2015). More than 50% of people develop PTE after severe TBI. According to the American Academy of Neurology, severe TBI is defined as the condition in which a person stays in coma for longer than 24 h post-injury or requires a neurosurgical intervention. Among those who develop epilepsy after severe TBI, nearly 40% experience their first seizure within 6 months of injury, 50–60% within a year and about 80% in the later years of life (Annegers et al., 1998; Agrawal et al., 2006; Pohlmann-Eden et al., 2006; Ding et al., 2016).

**FIGURE 1:**
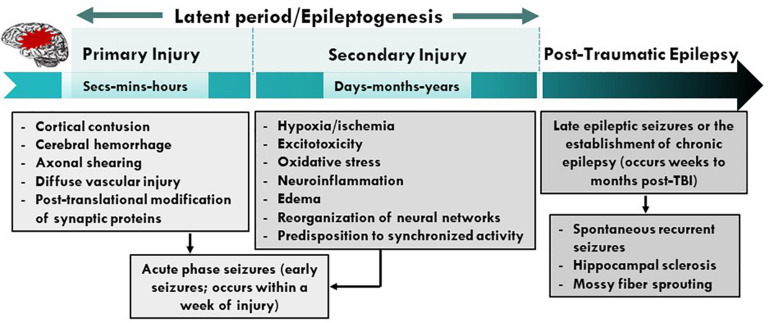
Pathophysiological basis of epileptogenesis following TBI. Complex and multifaceted events triggered during primary injury contribute to secondary injury. These primary and secondary events, which take place over months or years, represent a period of epileptogenesis. Changes such as post-translational modifications of synaptic proteins, reorganization of neural circuits and production and activation of neuroinflammatory molecules/pathways result in PTE. PTE is a progressive process which is a result of mossy fiber sprouting, hippocampal sclerosis, neuroinflammation, neurodegeneration and SRS. PTE, post-traumatic epilepsy; SRS, spontaneous recurrent seizures.

Traumatic brain injury is the third most common cause of all epilepsies and results from either direct (primary) or indirect (secondary) damage to brain parenchyma (Kaur and Sharma, 2018; Fordington and Manford, 2020). Trauma or brain injury results in both focal and diffuse injury to the central nervous system (CNS) that can trigger epileptogenesis (Shlosberg et al., 2010; Webster et al., 2017). Focal injuries usually cause contusion, hemorrhage, infarction, and necrosis, causing cortical scarring that effects synaptic plasticity and recovery. On the other hand, diffuse injury leads to axonal shearing, microvasculature damage, release of inflammatory mediators, and free radical overload (Greenfield et al., 2008; Mckee and Daneshvar, 2015). These injuries sabotage vulnerable neuronal populations and white matter tracts; and reactive gliosis that follow neuroinflammation (Wang et al., 2008; Lamar et al., 2014). Later, secondary injury mechanisms reorganize the neural circuits and disrupt brain homeostasis, with the degree of secondary damage largely depending on the severity of primary damage. A mild injury may deteriorate and remodel neural circuits to a lesser extent, whereas a severe insult not only reorganizes neural networks but also cause long-term degenerative changes that results in neuropsychiatric conditions, and cognitive and behavioral deficits (Burda et al., 2016; Ladak et al., 2019). For instance, the release of glutamate after severe head injury causes excitotoxic cell death via excessive calcium release, and generation of free radicals such as reactive oxygen and nitrogen species (ROS/RNS), which elicit an oxidative response against the mitochondria. Further, the recruitment of glial cells and peripheral immune cells (such as leucocytes and macrophages) aggravate the neuroinflammatory response by secreting cytokines. This enhanced proinflammatory response, combined with endothelial ROS, deteriorates the blood-brain barrier (BBB) integrity (Rosenfeld et al., 2012). These combined mechanisms of primary and secondary insults commence a vicious cycle of neurodegenerative events that persist for months to years, executing permanent degenerative changes in the brain (Figure 1). This review highlights cellular and molecular mechanisms that promote seizures, epileptogenesis and epilepsy after TBI. We also discuss the role of immune system, contribution of glial cells, long-term consequences of TBI and therapeutic strategies for managing PTE.

## Molecular Mechanisms of Post-Traumatic Epilepsy

### Hyperexcitability/Excitotoxicity and BBB Breakdown in TBI

#### Hyperexcitability/Excitotoxicity

After TBI, excitotoxicity in the brain is generally caused by an increase in extracellular glutamate. Under physiological conditions, glutamate is taken up by astrocytes and converted into glutamine which is then shuttled back to neurons as an alternative energy source (Dienel, 2014). However, excess glutamate overloads astrocytes’ capacity to remove glutamate from the extracellular space (ECS), triggering an enormous Ca^2+^ and Na^+^ influx and K^+^ efflux (Tehse and Taghibiglou, 2019). This resulting ionic imbalance depolarizes the postsynaptic cell membrane, causing a long-lasting increase in excitatory post-synaptic potential. Altered calcium signaling after TBI activates nitric oxide synthase (NOS), proteases, and lipases that trigger cell signaling cascades linked to excitotoxicity and cell death (Weber, 2012; Jarrahi et al., 2020). Elevations in nitric oxide (NO) levels interferes with mitochondrial bioenergetics leading to energy depletion, further adding oxidative stress in neurons. The changes in mitochondrial bioenergetics initiate the release of cytochrome-c- activating caspases, that cause inflammation-induced apoptosis (Adam-Vizi and Starkov, 2010; Rowley and Patel, 2013; Puttachary et al., 2015). Compromise to mitochondrial integrity after TBI elicits the release of ROS/RNS, which deteriorates membrane lipids, proteins, and DNA, and downregulates the expression of glutamate transporters such as GLT-1 and GLAST promoting cellular excitotoxicity (Trotti et al., 1998; Abdul-Muneer et al., 2015; Chen et al., 2020). TBI-induced ATP depletion cause loss of Na^+^/K^+^ ion concentration gradient across the plasma membrane due to dysfunctional Na^+^/K^+^-ATPase, and leads to excitotoxicity-induced cell stress (Lima et al., 2008). In addition to dysfunctional Na^+^/K^+^-ATPase induced excitotoxicity, cell death via lysis or apoptosis also releases cytoplasmic glutamate in ECS after TBI (Zhang et al., 2005). These two forms of glutamate release cause a continual domino effect of cellular excitability that elevates extracellular glutamate concentration in the injured brain.

Numerous *in vivo* studies on rodent models of TBI have reported an increased glutamate levels in the brain of injured mice, 1–2 days post-injury (Hinzman et al., 2010; Guerriero et al., 2015). *Ex vivo* studies on brain slices using extracellular field potential recordings have reported elevations in excitatory inputs and evoked synaptic connections between dentate granule cells with mossy fibers, when stimulated with glutamate photostimulation in controlled cortical impact (CCI) model (Hunt et al., 2010). Similar studies, using FRET-based glutamate sensors on hippocampal slices, also reported enhanced cortical excitability and glutamatergic signaling, and increased spread of perforant-path stimulation evoked depolarization in brain slices of CCI and weight drop animals, 2–4 weeks post-injury (Golarai et al., 2001; Cantu et al., 2015). These and other studies confirmed that increase in glutamate response after injury modulate neuronal microcircuits that correlates with an increase in epileptiform activity adjacent to the site of injury.

#### Blood-Brain Barrier Breakdown in TBI

**FIGURE 2:**
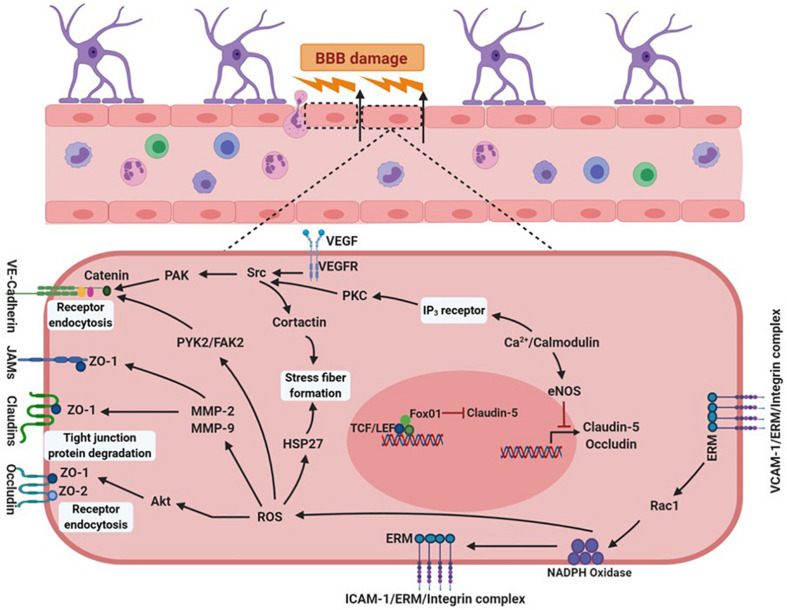
Vascular endothelial signaling after brain injury. In a brain inflicted with traumatic injury, inflammatory conditions stimulate signaling pathways in the cells of NVU. Cytokines TNFα and IL-1 released from blood cells activate receptors present on the vascular lumen near the sites of inflammation. These cytokines upregulate expression of selectins, chemokines, and integrin ligands like ICAM-1 and VCAM-1 on the surface of the endothelial cells, causing increased production of NADPH oxidase. Elevations in NADPH oxidase-mediated release of ROS degrades tight and gap junction proteins such as occludins, claudins and connexins, by generating excessive amounts of Akt and MMPs (MMP-2 and MMP-9). Oxidative stress enhances the production of FAK2, that damages adhesion molecules, cadherins. Alternatively, increased calcium inside the cell activates IP_3_ receptors resulting in increased PKC production, which in turn generates PAK via phosphorylation of Src kinase. PAK is also activated by Src phosphorylation via the activation of VEGF receptors. These events collectively damage cadherins compromising integrity of cellular junctions. Elevations in calcium levels also produces eNOS, which blocks claudin-5 and occluding transcription. These events cause BBB breakdown, allowing increased migration of peripheral immune cells into the central nervous system. NVU, neurovascular unit; ICAM-1, intercellular adhesion molecule 1; VCAM-1, vascular cell adhesion protein 1; MMPs, matrix metallopeptidase; FAK2, focal adhesion kinase 2. Figure created with BioRender.com and R&D Systems.

Blood-brain barrier disruption has a well-recognized role in the pathophysiology of CNS diseases; and understanding the anatomy and physiology of the neurovascular unit in health and disease is critical for advancing translational research into the clinics. Many studies demonstrated that BBB integrity is lost in CNS diseases such as meningitis, encephalitis, Alzheimer disease, Parkinson’s disease, multiple sclerosis, and epilepsy. Damage to the components of neurovascular unit (NVU) such as endothelial cells, after TBI, can impair BBB. Dysfunctional endothelial cell signaling and activation of the immune cell response stimulates the release of proinflammatory mediators, such as ROS, matrix metalloproteinases (MMPs), bradykinins, prostaglandins, cytokines, tachykinins, and excitatory amino acids (Paudel et al., 2019). The formation of intercellular adhesion molecule 1 and vascular cell adhesion protein 1/ERM complex with integrin, via Rac1, releases NADPH oxidase (enzyme involved in oxidative stress) in the endothelial cells generating ROS (Cerutti and Ridley, 2017; Jarrahi et al., 2020). Elevations in ROS levels stimulate the release of MMP-2 and 9 causing damage to tight and gap junction proteins such as occludins, claudins and connexin-43. A further rise in oxidative stress activates focal adhesion kinase, a non-receptor tyrosine kinase, and heat-shock protein 27, that results in receptor endocytosis and stress fiber formation within the cell (Hemphill et al., 2011; Cerutti and Ridley, 2017; Jarrahi et al., 2020). In addition, vascular endothelial growth factor stimulated increase in Src increases phosphorylation of VE-cadherins via serine/threonine-protein kinase, which results in receptor endocytosis. Concurrently, an increase in intracellular calcium activates calcium/calmodulin complex that generates endothelial nitric oxide synthase (eNOS). Rise in eNOS levels inhibits the transcription of claudin-5 and occludin, further increasing BBB permeability (Badaut et al., 2015; Andrews et al., 2016; Cerutti and Ridley, 2017) (Figure 2). An activation of inflammation and the immune response triggers a heightened neuronal response, stimulating neurotransmitter release from the endothelial cells via activation of the central-mediated hypothalamic-pituitary-adrenal axis (Licinio and Frost, 2000; Silverman et al., 2005; Burfeind et al., 2016). These deleterious events initiate multiple signaling transduction pathways, causing changes in BBB permeability and activation of signaling enzymes, such as kinases, to regulate calcium mobilization and gene expression. This affects the transport characteristics of proteins located on endothelial cells, promoting excitotoxicity (Dalal et al., 2020). Therefore, changes in BBB permeability and enhanced endothelial paracellular leak (due to tight junction protein modifications) alter the volume regulators that control BBB homeostasis. This alters tight junction proteins, leading to reorganization and remodeling of the cytoskeletal proteins disrupting brain homeostasis (Stamatovic et al., 2008; Burda et al., 2016).

**FIGURE 3:**
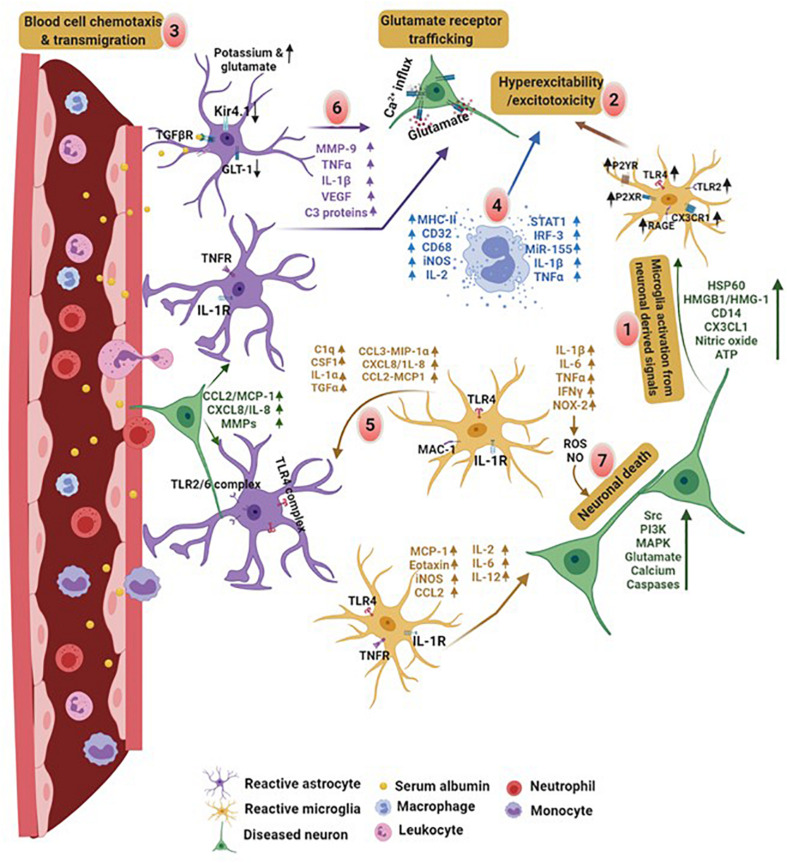
Comprised BBB integrity and neuro-glia crosstalk in PTE. Proinflammatory factors released by damaged neurons and glial cells initiate a vicious cycle of inflammation in the brain. **(1)** Brain injury results in an early activation of microglia and stimulate production of cytokines and expression of DAMPs associated receptors on their surface, leading to neuronal excitability. **(2)** Increased cellular hyperexcitability due to molecules released by reactive glia and neurons promote, **(3)** blood cell chemotaxis and transmigration into the brain. Disruption in BBB leads to brain infiltration of immune cells from the blood releasing **(4)** proinflammatory factors and promoting excitotoxicity. **(5)** Later, activated microglia release signals that bind to the receptors on astrocytes. **(6)** Serum albumin induced activation of astrocytes impairs potassium channel and glutamate transporter. In addition, activated astrocytes release proinflammatory factors which exacerbates neuronal hyperexcitability. **(7)** These factors collectively or on their own cause neurodegeneration. Cytokine mediated crosstalk between astrocyte-microglia and neuro-glia induces hyperexcitability and neuronal cell death. In contrast, molecules derived from neurons, such as HSP60, HMGB1, CD14, CX3CL1, and ATP, cause microglia activation leading to excitotoxicity. Monocytes and leukocytes also release cytokines at this point. This creates an activation cycle which causes release of additional inflammatory molecules triggering cytokine storm in the brain. Discharge of molecules like MHCII, complementary proteins, proinflammatory interleukins, iNOS, transcription factors and microRNAs by macrophages, cause excitotoxicity in neurons. These molecules promote neurodegeneration and initiate neuroinflammatory pathways lowering the seizure threshold. HSP60, heat shock proteins; HMGB1, high mobility group box 1; CD14, cluster of differentiation 14; CX3CL1, chemokine (C-X3-C motif) ligand 1; ATP, adenosine 5′-triphosphate; iNOS, inducible nitric oxide synthase. Figure created with BioRender.com (2020). Retrieved from https://app.biorender.com/biorender-templates.

After TBI, loss in BBB integrity is primarily due to the release of excitotoxic factors by injured neurons and activated glial cells. These factors drive blood cell chemotaxis and their transmigration into the brain. Enhanced leukocyte infiltration and invasion of CNS parenchyma generates a cytokine storm which induces neuronal injury. Infiltration of leukocytes also increases accumulation of intracellular fluid and capillary pressure causing turnover in the transendothelial volume. This can lead to traumatic brain edema exemplifying a transcytosis response to injury (Castejon, 1984; Scallan et al., 2010). Additionally, concurrent modifications take place in glial cells that drive morphological and molecular changes in order to attain reactive morphology. Increased proinflammatory secretions from neurons and reactive glial cells facilitate recruitment of additional immune cells, such as neutrophils and monocytes, from the periphery further modulating brain activity by increasing proinflammatory receptor expression on their surface. The binding of molecules released by neighboring glial cells and injured neurons cause activation of these receptors, exacerbating neuronal excitotoxicity (Medzhitov, 2008; Aronica et al., 2012; Burda and Sofroniew, 2014; Sanz and Garcia-Gimeno, 2020). Increased blood immune cell infiltration and dysfunctional neuro-glia crosstalk cause further rise in cytokine storm, therefore damaging BBB and its components (Figure 3).

##### Pericytes in traumatic brain injury

Traumatic brain injury can have deleterious effects on the neurovascular unit (NVU). Pericytes, an important component of NVU found in capillaries around the brain and other regions, play an important role in the maintenance of BBB, angiogenesis, regulation of blood flow and immune cell movement in the brain (Brown et al., 2019). After brain injury, dysfunctions in pericyte signaling results in the loss of pericyte–endothelium interactions, allowing easy passage for neurotoxins from the blood to enter brain. *In vivo* studies on the mouse model of TBI have reported reduced expression of pericyte markers, platelet-derived growth factor-B (PDGF-B), NG2 and CD13, 24 h post lateral fluid percussion injury (LFPI). Reduction in these markers also corresponds to a decrease in tight and gap junction proteins (Bhowmick et al., 2019). Alterations in these proteins cause increased water permeability in the brain due to a substantial changes in aquaporin (AQP4) expression around the perivascular region. These studies also reported higher expression of calcium binding protein and a reactive astrocyte marker, S100β, and neuron-specific enolase in the blood samples of TBI animals (Bhowmick et al., 2019). Using two different adult viable pericyte deficiency mouse strains with variable degrees of pericyte loss, Bell et al. (2010) demonstrated that pericyte loss during neurodegenerative conditions can influence brain capillary density, resting cerebral blood flow, blood flow responses to brain activation and blood brain integrity to serum proteins, and blood derived cytotoxic and neurotoxic molecules. Using *in vivo* multiphoton microscopy on mouse lines expressing PDGFRβ^+^ exclusively on pericytes, the same group further reported a significant reduction in the length of perfused capillaries that corresponded to a reduction in cerebral blood flow volume (Bell et al., 2010). BBB disruption after TBI causes dysfunction in pericyte-endothelium interaction and increase in toxic accumulation of serum derived proteins in the brain. Using time-lapse imaging of a low-molecular weight 40 KDa TMR-Dextran, studies have reported diminished brain capillary perfusion and changes in vascular permeability in PDGFRβ^–/–^deficient mice. Golgi histological analysis of neuronal structure and function revealed progressive loss of dendritic spines and significant structural abnormalities in CA1 region of hippocampus in PDGFRβ heterozygous mice at 8 and 16 months of age, supporting a crucial role of pericytes in neurovasculature (Bell et al., 2010). Studies on mouse models of TBI have shown diminished pericyte-endothelium interactions showing reduced oxygenation in ipsilateral and contralateral areas of the somatosensory cortex, as well as other regions of the brain, during early stages of TBI (Johnstone et al., 2014; Zehendner et al., 2015; Ichkova et al., 2020). Using acute brain slices and vascular staining, studies reported changes in neurovascular reactivity and morphological variations in the blood vessels of mice, 1 and 30 days post-injury (dpi). These changes reversed during early and late stages, revealing time-dependent alterations in the neurovasculature and dysfunction in oxygenation and vascular coupling (Ichkova et al., 2020). The results from these and other studies suggest that neurodegenerative changes develop following a primary vascular insult which impairs pericyte–endothelium interactions. Disruption in pericyte signaling alters brain microcirculation causing diminished brain capillary perfusion. This leads to chronic perfusion stress and cellular and molecular alterations of BBB, which includes, changes in transport functions of endothelium, loss of pericytes, decrease in cerebral blood flow, loss of vascular reactivity, changes in vascular morphology, alterations in glial metabolic rate and oxygen deficiency in tissues. These post traumatic brain injury events lead to cellular excitotoxicity and chronic neurodegeneration (Bell et al., 2010; Wu et al., 2020).

### Oxidative Stress and Neurodegeneration

#### Oxidative Stress

Mitochondrial dysfunction has long been recognized as a key source of oxidative stress in epilepsy. Emerging evidence suggests that acute seizures induce oxidative stress, and as a result of initial insult, the process of epileptogenesis begins to dominate the brain (Patel, 2004; Liang and Patel, 2006). During oxidative stress, deleterious changes in mitochondria include altered mitochondrial membrane potential, enhanced nicotinamide adenine dinucleotide phosphate (NADPH) production, impairment of electron transport chain complex 1, 3, and 4, rise in mitochondrial ROS, and mitochondrial DNA damage (Dexter et al., 1989; Cini and Moretti, 1995; Chuang et al., 2004; Kann et al., 2005; Chuang, 2010). These changes in mitochondrial activity cause progressive dysfunction, aligning with a common theme of epileptogenesis as a series of degenerative events that triggers a vicious cycle of oxidative stress and neurodegeneration, ultimately leading to PTE (Vezzani et al., 2011).

#### Free Radicals of Oxygen and Nitrogen

Free radicals are generated by oxidation and reduction reactions of electrons during hemolytic cleavage, when the bond is broken in such a way that the pair of electrons is shared equally by both the separating fragments. These separating fragments may carry one or more unpaired electrons, which makes free radicals highly reactive in nature. Free radicals are chemically unstable molecules that cause cellular and mitochondrial DNA fragmentation (Lobo et al., 2010; Cardenas-Rodriguez et al., 2013; Ozcan and Ogun, 2015). As a result of oxidative damage, alterations in morphological and functional properties of proteins and lipids takes place. This further impacts cellular and mitochondrial DNA, and cross-link base pairs and cause genetic mutations (Emerit et al., 2004; Waldbaum and Patel, 2010; Ramalingam and Kim, 2012). Free radical species of oxygen and nitrogen include superoxide anion, hydroxyl radical (OH), peroxyl and alkoxyl radicals, hydrogen peroxide (H_2_O_2_), peroxynitrite, nitroxyl anion, nitrogen dioxide and nitrate/nitrite (Cheeseman and Slater, 1993; Ozcan and Ogun, 2015; Puttachary et al., 2015). An excessive generation of these radicals within the cell causes oxidative stress.

#### Free Radical Production and Oxidative Stress

Oxidative stress is a biochemical state when an excessive production of ROS/RNS cause damage to the cell membranes and proteins, as well as to cellular and mitochondrial genomes (Cardenas-Rodriguez et al., 2013). Majority of oxygen and nitrogen-centered free radicals are generated from interactions between NO and molecular oxygen (O_2_). NO is produced from the substrate L-arginine, with the help of a co-factor NADPH and O_2_, and enzyme nitric oxide synthase (NOS). In the cytoplasmic membrane, superoxides are primarily generated by NADPH oxidase, after an electron transfer from NADPH to O_2_. These superoxides are also generated by the action of O_2_ on xanthine oxidase. Under normal physiological conditions, superoxides are converted into H_2_O_2_ by the action of superoxide dismutase, which is then broken down into water and oxygen (with the help of catalase and glutathione peroxidase). Degradation of superoxide dismutase promotes enhanced production of highly reactive peroxynitrites (ONOO^–^), a powerful oxidizing agent, which results in increased ROS production, DNA, proteins and lipids oxidation and loss of ion channel dysfunction (Halliwell, 1999; Puttachary et al., 2015). Increased superoxide in a cell cause oxidative burst promoting oxidative damage by exacerbating inflammation, enhancing redox signaling and proinflammatory gene regulation (Agledal et al., 2010). Moreover, impairment of catalase and peroxides promote formation of hypochlorous acid from H_2_O_2_ by reacting with Cl^–^, NO^2–^ and phenols. This causes cell death by destabilizing calcium homeostasis. Alternatively, H_2_O_2_ can also undergo Fenton and Haber–Weiss reaction to form OH radicals (a harmful free radical of oxygen with high reactivity and a short half-life) which results in proteins and lipid peroxidation, mitochondrial DNA damage and depletion of antioxidant enzymes (Bae et al., 2011; Puttachary et al., 2015).

The metabolic regulation and signaling of redox enzymes, such as NADPH oxidase, lipoxygenase and endoperoxide synthase is exceedingly altered after TBI. At basal levels, NADPH oxidase (NOX-2) is expressed widely in the brain where it plays an important role in learning, memory consolidation, innate immunity, phagocytic activity and apoptosis (Infanger et al., 2006; Aguiar et al., 2012; Eastman et al., 2020). However, under pathological conditions, such as in PTE, NADPH oxidase generates greater amounts of superoxide ions, triggering neuroinflammation and neurodegeneration, as evidenced by various animal models of TBI and chemoconvulsant-induced TLE (Ferreira et al., 2013; Angeloni et al., 2015; Ma et al., 2017; Eastman et al., 2020). For instance, Li et al. (2019) on the chemical induced brain injury model of mice, reported the accumulation of oxidative stress factors such as lipid ROS and 4-hydroxy-2-nonenal (4-HNE) adducts in the somatosensory cortex and hippocampal HT22 cells, 12–36 h post-injury. The same group also discussed the involvement of oxidative enzyme 12/15 lipoxygenase (12/15-LOX) associated ferroptosis in a trauma induced neuronal damage, that corresponds to reduced cell viability and glutathione peroxidase 4 activity in the cortex of mice and in hippocampal cultures (Li et al., 2019). 12/15 LOX plays an important role in modulating oxidative stress and increase post-traumatic seizures by generating oxidized phospholipids (Chinnici et al., 2005). In a study on the rat model of LFPI, Saraiva et al. (2012) demonstrated that an increased levels of thiobarbituric acid and protein carbonylation contents in the brain increased seizure and spiking activity, within a week after injury (Saraiva et al., 2012). These and other studies provide a strong evidence and the significance of synergistic interactions between the redox enzymes in maintaining TBI-induced oxidative stress. In addition, detrimental role of prostaglandin-endoperoxide synthase, such as cyclooxygenase (COX-2) have also widely been reported in various clinical and experimental models of TBI and epilepsy. COX-2 upregulates ROS by producing prostaglandins (specifically, F2 and H), and stimulate astrocytes to produce proinflammatory cytokines which signals for oxidative stress-mediated neuronal death (Madrigal et al., 2006; Hickey et al., 2007; Rojas et al., 2014). COX-2 also initiates inflammatory response in immune cells such as neutrophils and alters tissue homeostasis (Ricciotti and FitzGerald, 2011). Interactions between NOS and COX-2, after brain injury, can affect neocortical development by creating pathological milieu (Kaufmann et al., 1997). Studies on immature rats have reported enhanced COX-2 expression after TBI, that corresponds to an increased NOS and prostaglandin synthesis. Studies have shown that increased lesion size after TBI, corresponds with an increased COX-2 expression, that leads to impaired cognitive deficits in rats (Hickey et al., 2007). These studies demonstrate that the accumulation of oxidative stress factors, after TBI, cause increased cytokine levels, NO metabolites, oxidative enzymes, protein carbonylation contents, SRS and memory deficits over time- which altogether may lead to PTE (Table 1). Inhibition of these enzymes have been shown to prevent cognitive deficits, motor dysfunctions, cerebral edema, cerebral perfusion rate, neurodegeneration and neuroinflammation, in many clinical and animal models (Madrigal et al., 2006; Zhang et al., 2012; Ferreira et al., 2013; Liu et al., 2016; Li et al., 2019). Therefore, targeting these molecules can provide neuroprotection against TBI-induced epileptogenesis.

**TABLE 1:** Biomarkers of TBI-induced epileptogenesis.

Experimental model	Specie, age, strain	Injury mechanism	Biomarkers analyzed	Time-points markers observed (post-TBI)	Region/s analyzed	Effects on brain physiology/mechanism/outcome	References
**Lateral Fluid Percussion Injury**	Rat, P32–35, Sprague Dawley	10 ms pressure pulse of 3.75–4 atm	GFAP; Cellular necrosis; Neocortical hyperexcitability; Epileptiform activity; SRS	• Gliosis and cellular necrosis: 6–16 weeks • Cortical hyperexcitability: 8–10 weeks • Epileptiform activity: 2–10 weeks • SRS: 2–8 weeks	Frontal-parietal and parietal-temporal neocortex; Thalamus	• Intense glial reactivity and neuronal depletion in neocortex and thalamus • Neocortical hyperexcitability in frontal, parietal I and II areas	D’Ambrosio et al., 2004
**Lateral Fluid Percussion Injury**	Rat, 305–390 g, Sprague Dawley	21–23 ms pressure pulse of 2.6–3.3 atm	Neuronal loss; MFS; Behavioral seizures; Epileptiform activity; SRS	• Hippocampal cell loss and MFS: 10–12 months • SRS: 8–52 weeks	Frontal and parietal cortex; Hippocampus	• Ipsilateral loss of dentate hilar neurons • Enhanced MFS in ipsilateral hippocampus • Increased behavior seizure severity • 50% animals developed epilepsy after severe injury	Kharatishvili et al., 2006
**Rostral parasagittal FPI**	Rat, P33–35, Sprague Dawley	10 ms pressure pulse of 3.25–3.5 atm	GFAP; Neuronal loss; Thalamic calcification; CA3 hyperexcitability; SRS	• Gliosis and neuronal loss: 2–4 weeks and 7 months • SRS: 2–8 months	Hippocampus; Thalamus; Temporal neocortex; Frontal-parietal cortex	• Increased glial immunoreactivity and neuronal depletion • Progressive shrinkage of ipsilateral hippocampus (hippocampal atrophy) and temporal neocortex with loss of laminar features • Increased bilateral seizures in hippocampus and cortical discharges over time	D’Ambrosio et al., 2005
**Controlled Cortical Impact/Lateral Fluid Percussion Injury with PTZ**	Mice, 10–11 weeks, C57BL/6S	CCI: Cortical compression at 0.5 mm depth at 5 m/sec velocity and 100 ms duration; FPI: 21–23 ms pressure pulse of 2.9 atm; 50 mg/kg PTZ (i.p.) 6 months post-CCI or FPI	Cortical contusion/lesion; MFS; Hippocampal neurodegeneration; Electrographic activity; SRS	• Cortical contusion, hippocampal neurodegneration and MFS: 6–9 months • Epileptiform discharges and SRS: 6–9 months	Frontal Cortex; Hippocampus	• Cortical lesion injury extended through all layers of cortex • Higher hippocampal neurodegeneration in granule cell layer, hilus, CA3 and CA1 • MFS more apparent septally than temporally • Increased epileptiform discharges, seizure susceptibility and SRS	Bolkvadze and Pitkänen, 2012
**Controlled Cortical Impact**	Mice, 8 weeks, CD-1	Cortical compression at 2 mm depth at 5 m/sec velocity and 100 ms duration	phospho S6; 4EBP1; STAT3; FJB; MFS; SRS	• phospho S6: 3, 6, 24 h, 3 days, 1 week, 2 weeks • 4EBP1: 3 days • STAT3: 6 h, 3 days • FJB: 3 days • MFS: 5 and 16 weeks • SRS: 10–16 weeks	Neocortex; Hippocampus	• Hyperactivation of mTORC1 pathway • Increased neuronal degeneration and MFS • Increased PTS frequency during early phases of disease progression	Guo et al., 2013
**Lateral Fluid Percussion Injury**	Rat, 8–9 weeks, Long-Evans	Percussion wave of 2.3 atm	GFAP; GLT-1; SRS	• Gliosis and GLT-1: 7 days • SRS: 12 weeks	Neocortex	• Suppression of GLT-1i • Increased GFAP expression and PTS frequency	Goodrich et al., 2013
**Fluid Percussion Injury with PTZ**	Rat, 250–300 g, Wistar	10–15 ms pressure pulse of 3.53 atm; 35 mg/kg PTZ (i.p.) 4–8 days post-TBI	TBARS; Protein carbonyl content; Na+-K+-ATPase activity; Early seizures	• TBARS and carbonyl content: 4 and 8 days • Na+-K+-ATPase activity: 3 and 7 days • Early seizures: 4–8 days	Parietal CTX	• Increased oxidative damage due to lipid and protein oxidation • Increased seizures and spiking activity	Saraiva et al., 2012
**Lateral Fluid Percussion Injury**	Rat, 305–390 g, Sprague Dawley	21–23 ms pressure pulse of 2.64–3.11 atm	Cortical lesion; FJB	• Cortical lesion: 12 months • FJB: 14 days	Cortex; Hippocampus	• Extensive degeneration and atrophy in injured cortex • Reduced cortical volume	Kharatishvili and Pitkänen, 2010
**Human sTBI**	Males, 18–65 years old	Severe TBI with Glasgow Coma Scale Score 4–8	GFAP; IL-6; S100β; NSE; TNFα; Estrogen; Progesterone	• Gliosis and IL-6: 8 h and 1 week • NSE: 1 week	Serum	• Increased gliosis and IL-6 over time in patients with severe TBI • High GFAP and IL-6 levels	Raheja et al., 2016
**Human TBI**	Males and females, 1 month- 13 year old	Based on lesion area and other demographic and clinical features	HMGB1; IL-1β; S100β; GFAP; AACT; Epileptiform discharges	• HMGB1, IL-1β, S100β & gliosis: within 24 h and 1 week after seizure onset • Epileptiform discharges: 6, 12, and 18 months	Serum	• Higher HMGB1, IL-1β, S100β and gliosis; • Abnormal EEG with epileptiform waves associated with increased HMGB1 and IL-1β levels	Zhu et al., 2018
**FeCl_3_-induced injury**	Male, 18–22 g, C57BL/6J	Stereotaxic injection of 50 mM FeCl_3_ in somatosensory cortex	Lipid ROS; 4-HNE adducts; PTGS2; GPX4; 12/15 LOX	• Lipid ROS, 4-HNE adducts, PTGS2, GPX4, 12/15 LOX: 12–36 h	Somatosensory cortex; Hippocampal HT22 cells	• 12/15-LOX associated ferroptosis dependent Fe-Cl_3_-induced neuronal damage • Reduced cell viability & GPX4 activity • Increased ferroptotic inducers (lipid ROS, 4-HNE and PTGS2 mRNA)	Li et al., 2019
**Controlled Cortical Impact**	Rat, 2–11 month, Sprague Dawley	Cortical compression at 2.8 mm depth at 4 m/s velocity and 100 ms duration	GABA_*A*_R α1, α4, γ2 & δ subunits; NR2B; GluR1; HSP70 and HSP90; NeuN; SRS	• GABA_*A*_R α4 and δ subunit, NR2B and HSP70: 5–9 months • SRS: 3–9 months	Cerebral cortex; Hippocampus	• Reinforced hyperexcitability and seizure susceptibility after GABA_*A*_R modulation • Altered NR2B, HSP70 and GluR1 expression • Tissue loss and necrotic cavity formation in right ipsilateral hemisphere • Morphological changes in ipsilateral hippocampus	Kharlamov et al., 2011
**Controlled Cortical Impact**	Mice, 8–10 weeks, CD-1	Cortical compression at 1 mm depth at 5 m/s velocity and 200 ms duration	AQP4; Kir4.1; GFAP; SRS	• AQP4 and Kir4.1: 30 and 60 days in cortex • Gliosis: 14, 30, 60, and 90 days • SRS: 14–90 days	Frontal cortex; Hippocampus	• Mislocalized and dysregulated perivascular AQP4 associated astrocytic swelling • Decreased ECS and increased ephatic interactions	Szu et al., 2020
**Fluid Percussion Injury with/without KA**	Rat, 297 g, Sprague Dawley	Pulse pressure of 2.3 atm	Cell loss; 2DG/FDG; ^14^C-AIB	• Cell loss: 7 days • 2DG/FDG (glucose metabolism): 75 min • ^14^C-AIB (BBB permeability): 70 min	Hippocampus; Plasma	• Increased ipsilateral ICMRglc after double insult paradigm • Enhanced regional BBB permeability • Hippocampal cell loss and damage	Zanier et al., 2003
**Lateral Fluid Percussion Injury**	Rat, 4 weeks, Wistar	Pulse pressure of 2.0–2.2 atm	fEPSP from DG cells; GluA1 and GluA2; MAP2; GFAP; IBA1; CD45; CD3; CD4; GR-1; OX42; SRS	• MAP2, gliosis and IBA1: 24 h • DG hyperexcitability, GluA1 and GluA2: 7 days • CD45, CD3, and CD4: 5–6 days • SRS: 12–15 weeks	Hippocampus; Brain slices; Primary hippocampal neurons	• DG granule cell AMPAR based network excitability • Increased seizure susceptibility by TLR4 signaling in neurons • Neuronal loss	Korgaonkar et al., 2020
**Lateral Fluid Percussion Injury**	Mice, 8 weeks, C57BL/6J	12–16 ms pressure pulse of 1.5 atm	CD3e; CD4; CD19; CD8; MHC II; CLIP; FJC; GFAP	• CLIP: 24 h • FJC and gliosis: 3 days post-injury	Parietal CTX; Brain leukocytes; Intestinal lymphocytes	• Enhanced astrocytic response in perilesion cortex • Increased CLIP-dependent neurodegeneration via CD74 cleavage • Increased brain immune cell infiltration after MIF-binding • CD74 and MIF-dependent astrocyte activation	Newell-Rogers et al., 2020
**Controlled Cortical Impact with/without PTZ**	Rat, 250–280 g, Wistar	Cortical compression at 2 mm depth at 4.5 m/s velocity and 150 ms duration; 30 mg/kg PTZ (i.p.) 24 h post-TBI	Brain contusion; IL-1β; TNF-α	• Brain contusion • IL-1β and TNF-α : 4 and 12 h	Hippocampus	• Accelerated rate of kindled seizure acquisition • Increased TNF-α and IL-1β overexpression • Increased neuroinflammation and neural damage	Eslami et al., 2015
**Controlled Cortical Impact with electrical kindling**	Rat, 9 weeks, Wistar	Cortical compression at 2 mm depth at 4.5 m/s velocity and 150 ms duration; Electrical kindling (50 μA at 5-min intervals).	Cortical lesion volume; TNF-α	• Cortical contusion and TNF-α: 24 h	Parietal cortex	• Increased seizure duration directly correlated to increased TNF-α levels	Hesam et al., 2018
**Parasagittal Fluid Percussion Injury with PTZ**	Rat, 294–384 g, Sprague Dawley	Pulse pressure of 1.9–2.1 atm; 30 mg/kg PTZ (i.p.) 2 weeks post-TBI	Cortical lesion volume; NeuN	• Cortical contusion and cortical and hippocampal neuronal cell loss: 2 weeks	Cortex; Hippocampus	• Increased cortical contusion and volume • Neuronal depletion in parietal cortex and hippocampus	Bao et al., 2011
**Controlled Cortical Impact with PTZ**	Mice, P21, C57BL/6J	Cortical compression at 1.2–1.73 mm depth at 4–4.5 m/s velocity and 150 ms duration; 30–50 mg/kg PTZ (i.p.)	Cortical lesion volume; IL-1R1; IL1-1β; GFAP; Vimentin; ZnT3; NeuN; IBA1; SRS	• Tissue deformation and volumetric loss: 6 months • ZnT3: 2 weeks and 3 months • IL-1β: 2–12 h and 1–14 days • IL-1R1 and Vimentin: 1 day • GFAP: 1 day, 14 days, and 6 months • SRS: 4–5 months	Cortex; Hippocampus; Corpus Callosum; Serum	• Abnormal hippocampal MFS at lesion epicenter • Robust hippocampal gliosis • Long-term structural reorganization in DG • IL-1R/IL-1β mediated post-traumatic alterations in network excitability • Cortical tissue loss	Semple et al., 2017
**Closed Head Injury with Electroconvulsive Shock**	Mice, 20–25 g, CD-1	2 mm steel tip impounder at 6 m/s velocity and impact depth 3.2 mm	GFAP; S100β; IBA1; NeuN; MT-1 and MT-2	• Gliosis, S100β, IBA1 and MTT: 8 days • GFAP and S100β: 14 days	Hippocampus	• Increased neurobehavioral impairment due to increased gliosis and metallothionein levels • Greater neurological injury after enhanced astrocytic release of MTT • Increased seizure susceptibility associated with greater glial activation and cytokine response	Chrzaszcz et al., 2010
**Closed Head Injury with PTZ**	Mice, 6–8 weeks, C57BL/6J	5–7 mm impactor at 7.14 m/s velocity during 100 ms period; 10 mg/kg PTZ (i.p.) 3 days post-TBI	GABA potential; NKCC1; KCC2; TGF-β2; NeuN; GFAP	• Reversal potential of GABA_*A*_ currents: 3 days • NKCC1 and TGFβ: 3 h, 1, 3, and 7 days • Gliosis; 3 days	Cortex; Hippocampus; Cortical Brain Slices	• Astrocytic TGFβ involved in neuronal upregulation of NKCC1 • Increased early PTS through NKCC1 mediated hyperexcitability • Increased seizure severity by TGFβ mediated NKCC1 expression	Wang et al., 2017
**Weight Drop with PTZ**	Rats, 250–400 g, Sprague Dawley	20 g weight dropped from 20 cm height; 30 mg/kg PTZ (i.p.) 15 weeks post-TBI	Neuronal loss and degeneration; FJB; pEPSP	• Cell loss: 2–27 weeks • Neurodegeneration: 1–5 days and 2 and 8 weeks • MFS: 15 weeks • DG excitability: 2–3 weeks and 14–15 weeks	Somatosensory cortex; Hippocampus; Brain Slices	• Gross cell loss and neurodegeneration in hippocampal CA3 over time • Atrophy of ipsilateral hilus and reproducible damage to somatosensory cortex • Long-term persistent DG hyperexcitability	
						• Increased spread of depolarization evoked by perforant-path stimulation in slices	
						• Bilateral development of MFS with unilateral loss of bilaterally projecting hilar neurons	Golarai et al., 2001
**Controlled Cortical Impact**	Mice, 28–35 g, CD-1	Cortical compression at 1 mm depth at 3.5 m/s velocity and 400 ms duration	Glutamate stimulation/EPSC; MFS; Dentate granule cell excitation; SRS	• MFS and EPSC: 8–12 weeks • SRS: 6–10 weeks	Hippocampus; Brain Slices	• Increased DG excitatory input • Evoked synaptic connections between granule cells with MFS in slices	Hunt et al., 2010
**Controlled Cortical Impact with/without PTZ**	Mice, 12–14 weeks, C57BL/6J	Cortical compression at 0.5 mm depth at 5 m/s velocity and 100 ms duration; 30 mg/kg PTZ (i.p.) 15 weeks post-TBI	Cortical degeneration and lesion; MMP-9; Epileptiform activity	• Cortical degeneration: 1, 7, 14, and 30 days • Cortical lesion: 14 weeks • MMP-9: 10–60 min, 2–6 h, 1–30 days • Epileptiform activity: 12 weeks	Somatosensory cortex; Hippocampus	• Somatosensory cortex degeneration and long-term motor function • MMP-9 mediated structural changes and increased seizure susceptibility over time • MMP-9 dependent increased lesion volume	Pijet et al., 2018
**Fluid Percussion Injury with PTZ**	Mice, 23–28 g, C57BL/6J	12–16 ms pressure pulse of 1.5–1.7 atm; 30 mg/kg PTZ (i.p.) 30 days post-TBI	Cortical lesion; Neurodegeneration; GFAP; IBA1	• Cortical lesion, neurodegeneration, gliosis: 1, 3, 7, and 30 days	Cortex; Hippocampus	• Glial scarring and robust glial response early after injury • Increased neurodegeneration associated with increased gliosis • Persistent necrosis in the region surrounding the impact zone	Mukherjee et al., 2013
**Controlled Cortical Impact**	Mice, 25–30 g, CD-1	Cortical compression at 0.5–1 mm depth at 3.5 m/s velocity and 400 ms duration	MFS; EPSP; SRS	• MFS: 7 and 42–71 days • DG excitability and SRS: 42–71 days	Hippocampus; Brain Slices	• Axonal reorganization at early and later stages of injury proximal to the lesion • Spontaneous epileptiform activity in slices with robust MFS • Interval-specific changes in paired-pulse ratio associated with MFS • Unprovoked seizures due to injury-induced structural changes	Hunt et al., 2009
**Weight Drop with/without Pilocarpine**	Mice, 8 weeks, C57BL/6J	50 g weight dropped from 80 cm height; 250–350 mg/kg pilocarpine (i.p.) 24 h post-TBI	Thrombin; IL-1β; TNF-α; HPRT; Factor X	• Thrombin; IL-1β; TNF-α; HPRT; Factor X: 24 h	Hippocampus	• Enhanced thrombin activity related to PTS • Increased inflammatory markers, HPRT and Factor X, correlated with seizure severity	Ben Shimon et al., 2020

SRS, spontaneous recurrent seizures; MFS, mossy fiber sprouting; PTS, post-traumatic seizures; ECS, extracellular space. * Only the time-points of biomarkers with increased activity after TBI are described in the table.

#### Neurodegeneration

**FIGURE 4:**
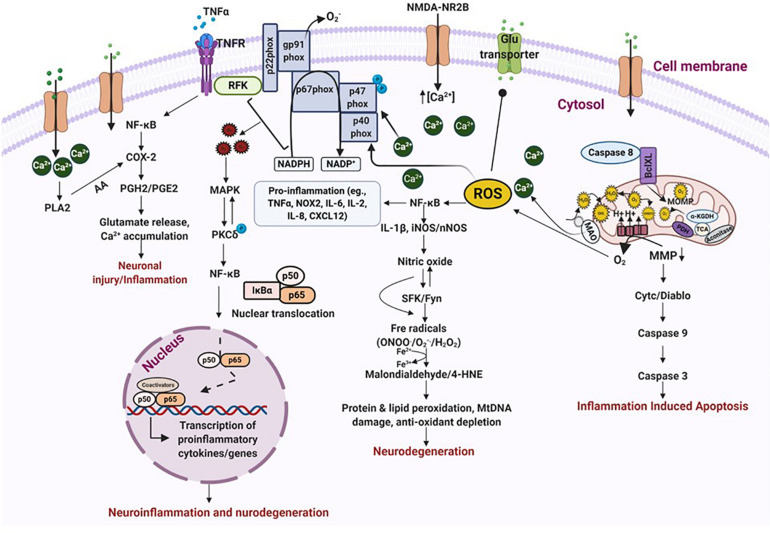
Oxidative stress leads to neurodegeneration and neuroinflammation. ROS/RNS incites a multitude of different events that leads to oxidative stress and neurodegeneration. Binding of TNFα to its receptor triggers activation of transcription factors which stimulate the release of prostaglandins and cause neuronal injury via excessive calcium release. An inflammatory stimulus also activates NOX enzymes, either on its own or via TNFα, which produces exorbitant amounts of free radicals of oxygen and nitrogen. This results in peroxidation of proteins and lipids, DNA damage and depletion of anti-oxidant proteins, ultimately causing neurodegeneration. These events also impair mitochondrial bioenergetics leading to inflammation induced apoptosis. Increased calcium influx, due to NMDAR trafficking, also triggers NOX activation and blocks glutamate transporters causing excitotoxic cell death. Increased accumulation of free radicals activates MAP kinase which in turn activates PKCδ. PKCδ phosphorylation promotes transcription of proinflammatory proteins either directly or through NF-κB activation. All these events finally resolve into neuroinflammation and neurodegeneration. ROS/RNS, reactive oxygen/nitrogen species; NOX, nicotinamide adenine dinucleotide phosphate oxidase; MAP, mitogen-activated protein kinase; PKCδ, protein kinase C delta. Figure created with BioRender.com.

Depending on the molecular mechanisms affected, neuronal cell death in TBI is classified as either physiologic or excitotoxic. Physiologic cell death is due to injuries that initiate cellular events such as mitochondrial swelling and nuclear membrane/cytoplasm rupture, whereas, excitotoxic cell death occurs a few hours after injury and causes chromatin agglutination and DNA fragmentation, but maintains an intact nuclear membrane (Stoica and Faden, 2010; Ladak et al., 2019). These intrinsic forms of cell death are primarily regulated by calcium release and enzyme-based regulators such as phospholipases, proteases, endonucleases, caspases, death-inducible complexes and pro-apoptotic proteins (Kögel and Prehn, 2000-2013; Broker et al., 2005; Raja et al., 2018). After TBI, the release of caspase-3 and caspase-12 disrupts the balance between pro-apoptotic and anti-apoptotic proteins, drawing the cell toward neurodegeneration and inflammation-induced apoptosis (Knoblach et al., 2002; Li and Yuan, 2008). Caspase-3 cleaves a specific serine-threonine protein kinase called PKCδ, causing its phosphorylation and activation. The activation of the NOX enzyme complex, either on its own or via TNFα, also increases PKCδ production. PKCδ trips the MAP kinase cascade, which allows NFκB to translocate into the nucleus, and transcriptionally activate proinflammatory genes (Sharma et al., 2018). PKCδ also regulates NOS expression and stimulates its release from reactive microglia and neurons, promoting lipid peroxidation by producing 4-HNE and malondialdehyde from hydroxyl radicals via a Fenton reaction (Puttachary et al., 2015; Sharma et al., 2018). 4-HNE impairs astrocytic proteins, such as glutamate transporter (GLT-1) which enhances free glutamate in the ECS. Free glutamate binds to NMDAR, causing NMDAR trafficking and calcium overload, free radical production, activation of gp91^*phox*^ (heme-binding subunit of NADPH oxidase) and, ultimately, cell-membrane protein degradation and cell death (Reyes et al., 2012; Pecorelli et al., 2015; Sharma et al., 2018). These events are progressive in nature and drive long-term neurodegenerative changes in the brain over time (Figure 4).

Physiological and structural evidence of dendritic loss, modulation of spine density and hippocampal sclerosis have widely been associated with increased seizure susceptibility after TBI (Golarai et al., 2001; Gao et al., 2011; Winston et al., 2013). There are numerous reports on the unilateral or bilateral loss of neurons in hilus and CA3 of hippocampus, progressive mossy fiber sprouting (MFS) in the inner molecular layer of DG and hyperexcitability in DG circuitry, several weeks after TBI (Lowenstein et al., 1992; Diaz-Arrastia et al., 2000; Golarai et al., 2001)-observations that were consistent with human PTE cases (Diaz-Arrastia et al., 2000). EEG and MRI studies on patients with intractable epilepsy, who suffered TBI, showed dysfunctions in temporal lobe as characterized by increased epileptiform spiking, dendritic spine remodeling, reactive gliosis and poor neuropsychologic response. These morphological changes in hippocampus were associated with MFS and hippocampal sclerosis (Diaz-Arrastia et al., 2000). Numerous studies on the rodent models of TBI have reported a strong association between dentate granule cell hyperexcitability and enhanced MFS with hippocampal sclerosis. These studies also demonstrated intense glial reactivity, DG hyperexcitability and neuronal loss in hilus of DG (Golarai et al., 2001; Kharatishvili et al., 2006; Hunt et al., 2009, 2010). Long-term persistent hyperexcitability in DG cause alterations in hippocampal pyramidal cell dendrites, that leads to reduction in spine density or spine loss (Jiang et al., 1998). Abnormalities in dendritic spines promote hyperexcitable circuits which directly influences neuronal excitability. The changes in number and morphology of spines are related to alterations in LTP and LTD, which can have a significant effect on the cognition (Wong and Guo, 2013). In PTE patients, the loss of dendritic spines has been observed in the pyramidal layers of hippocampus and in the granule cell layer of DG (Isokawa and Levesque, 1991; Wong, 2005). Dendritic atrophy, arborization, changes in dendritic length and even varicose swelling of dendrites were reported in some cases (Multani et al., 1994; Isokawa, 1998; Wong, 2005). Animal studies have not only provided a strong evidence for dendritic alterations after TBI, but also provided insights into the cellular and molecular mechanisms involved in such changes. It has been reported that changes in the neural circuits after TBI, and during early post-traumatic seizures can cause spine remodeling due to increased MMP-7 and 9 through NMDA-mediated receptor activation (Bilousova et al., 2006; Pijet et al., 2019). This alters neuromodulation resulting in excitotoxicity-induced neuronal death in the brain (Wong, 2005). For instance, experiments with GABA antagonist on hippocampal slice cultures revealed an increased spine loss in CA3 layer of hippocampus. This was partially reversed by an application of glutamate antagonist. Furthermore, application of glutamate agonist also caused loss of these spines due to NMDA-induced glutamate excitotoxicity due to activation of calcium-dependent enzymes, which degraded cytoskeletal structures (Müller et al., 1993; Jiang et al., 1998). Therefore, these studies strongly supports the role of NMDA receptors in dendritic spine loss and abnormalities that can be reversed using NMDAR modulators, suggesting the role of glutamate excitotoxicity in dendritic spine remodeling after TBI.

Numerous studies point to immunoregulatory molecules as master regulators of inflammation after injury. In cases of severe traumatic brain injuries, immunoregulators activate multiple signaling pathways that drives chronic microglial and immune response, and cause neurodegeneration (Loane et al., 2014). Interferons (IFNs) are among those pleiotropic signaling protein molecules that play a significant role in promoting neuroinflammation and neurodegeneration following PTE. IFNs are potent immune system activators and can act in an autocrine fashion to induce type I-IFN-driven inflammation and disease (Trinchieri, 2012; Uggenti and Crow, 2018). Type I IFNs play an important role in microglial activation and neurodegeneration, especially in the aging brain; and neutralization of such interferons alleviates cognitive deficits and slows down aging (Baruch et al., 2014). Recently, interest has been developed in identifying the DNA sensors responsible for IFN activation. An example of one such sensor is the cGAS-STING pathway. cGAS belongs to the nucleotidyltransferase family that activates STING by binding to DNA, which induces enormous amounts of type I IFN, driving neurodegeneration (Abdullah et al., 2018; Uggenti and Crow, 2018). In their CCI model, James Barret’s group recently reported increased cGAS and STING levels in the brain of juvenile mice, 3 days post-TBI. Using gene expression studies, they further reported enhanced mRNA expression of IFN-β and interferon regulatory factors such as IRF1, IRF4, and IRF7- factors that regulate amplification of type I IFNs in microglia. These studies show that microglia expressing high levels of IFNAR, following TBI, achieve reactive morphology and activation, and can prove to be a crucial target for IFNAR related diseases. Higher IFNAR expression subsequently increases production of TNF-α, NOX2, CCL5, and IL-1β mRNA, promoting cell death by driving synaptic and dendritic loss in neurons. In contrast, knocking out IFNAR and IFN-β has been shown to reverse these effects in the cortex and hippocampus of the mice (Karve et al., 2016; Barrett et al., 2020). Further, through behavioral studies, Barrett et al. (2020) demonstrated that knocking out *IFN*-β gene significantly improves motor and cognitive performance in the experimental subjects (Barrett et al., 2020). Moreover, several studies suggest that targeting IFN can prevent lesion volume, increase neuronal density, reduce the cytokine storm, decrease microglial activation and leucocyte infiltration, and limit neurodegeneration in the brain (Biliau, 2006; Mathur et al., 2017; Ta et al., 2019). Therefore, therapies targeting IFNAR can prove to be beneficial in treating TBI-associated neurological conditions. These therapeutic strategies can include using caspase inhibitors; cyclic dipeptides (to slow down cytochrome *c* release); use of pharmacological compounds (blocks cell cycle activators); progesterone and erythropoietin treatment (for edema and proinflammatory cytokine release); and statins (for governing Akt and slowing down microglial activation) (Ucciferri et al., 2007; Tayel et al., 2013; Zhu et al., 2013; Dejager et al., 2014).

### Neuroinflammation

Neuroinflammation in the brain is triggered by factors such as microbial infections, accumulation of toxic metabolites, traumatic brain and spinal cord injury, and tissue damage and malfunction. Acute inflammation, after TBI, activate molecules and signaling mechanisms that attempts to restore the body’s disrupted equilibrium by balancing inflammatory and resolution pathways. If these events are not controlled in time, they progress into a chronic stage, eliciting deleterious effects on the brain. The key molecules that regulate inflammation at this stage are granulocytes, platelets, prostaglandins, and cytokines released by lymphocytes, macrophages, microglia and stressed neurons. Their secretions cause intracellular modifications to recreate an unstable cellular microenvironment that disrupts cellular and molecular communications between cells (Herz et al., 2017; Davies et al., 2019; Scanlon, 2019).

Numerous studies on rodent models of epileptogenesis have reported on the post-TBI role of inflammatory mediators, prostaglandins and cytokines IL-1β and TNFα, in the hippocampus and other regions of the brain (Patel et al., 2017; Serhan, 2017). IL-1β, an immune cell mediator and IL-1RI ligand, has been associated with modulation of various neurological functions and in diseases. IL-1β increases NMDAR-mediated calcium release through the activation of Src family kinases (SFKs) (Viviani et al., 2003; Salter and Kalia, 2004). NMDAR are regulated by SFKs, especially by Fyn (Salter and Kalia, 2004). Substantial evidence suggests the link between increased IL-1β-NMDAR-SFK interactions in numerous neurological conditions influencing neuronal functions and enhancing neuronal excitability (Vezzani et al., 1999; Fogal and Hewett, 2008). Studies on hippocampal neurons have demonstrated that neurons exposed to IL-1β exhibit greater glutamatergic excitation and calcium release through NMDAR component, which induces excitotoxic cell death (Viviani et al., 2003). Interaction of IL-1β with IL-1R results in the recruitment of adaptor protein MYD88, which further recruits TRAF6 or IRAK I and II. The MYD88-TRAF6/IRAK I and II complex phosphorylates MAP kinase, causing NF-kB translocation into the nucleus, promoting transcription of proinflammatory genes (O’neill, 1995; Vezzani et al., 2011; Lalitha et al., 2018). These proinflammatory genes are primarily involved in cell death and survival, reorganization of molecular networks, plasticity, synaptogenesis and aberrant neurogenesis- events that takes place simultaneously with epileptogenesis (Vezzani et al., 2011). Activation of IL-1β/IL1R also promotes release of TNFα from astrocytes and glial cells. In contrast, toll-like receptor (TLR) activation stimulates TNFα expression as has been demonstrated in many experimental models of TBI (Yu and Zha, 2012; Shi et al., 2019). TNFα modulates neuronal excitability perhaps by internalizing inhibitory GABA_*A*_ receptors (Stellwagen et al., 2005; Stück et al., 2012; Pribiag and Stellwagen, 2013). TNFα binding to its receptor activates the TRADD complex and PI3 kinase, resulting in NF-κB activation modulating apoptosis and inflammation (Ermolaeva et al., 2008; Ting and Bertrand, 2016; Holbrook et al., 2019). TNFα activation also increases COX-2 production in response to injury, which is followed by an increase in PGE2 synthesis. Activation of these events cause glutamate accumulation and increases calcium load in the cell exacerbating neuroinflammation (Figure 4).

IL-1β and TNFα are undoubtedly the most well studied and widely known mediators of inflammation following TBI. Exuberant amount of work is underway, in both animal and human models, to target these molecules and prevent neurological outcomes related to TBI. For instance, in a mouse model of blast-injury, IL-1β antagonist Anakinra, has been shown to reduce gliosis, retinal degeneration and neuronal dysfunction (Evans et al., 2020). Another IL-1β synthesis inhibitor, VX-765, delayed seizure onset, duration and the number of SRS in chemoconvulsant induced experimental model of epilepsy (Maroso et al., 2011). In a study on TNFα inhibitors, C7 and SGT11, on a mice model of midline FPI, Rowe et al. (2018) reported significant improvements in cognitive deficits and sensorimotor function tasks (Rowe et al., 2018). Therefore, these studies provide strong evidence on the roles of IL-1β and TNFα inhibitors in modulating TBI-induced inflammation, and improving neurocognitive deficits, linked to TBI.

The role of prostaglandins in the animal models of TBI and in epileptogenesis is well known. Prostaglandins are produced by the action of COX-2 on arachidonic acid, which can be converted into five different prostanoids by the action of specific enzymes, depending on cellular conditions and their requirements. Prostanoids activate 11 receptors that primarily play a role in smooth muscle relaxation and contraction. Depending on the type of receptors and ligands activated, prostaglandins can play a significant role in various physiological and pathological conditions (Jiang et al., 2013; Rojas et al., 2014; Du et al., 2016; Eastman et al., 2020). Numerous studies showed high concentrations of prostaglandins in the brains of human patients and animals with temporal lobe epilepsy (TLE) (Takemiya et al., 2006; Jiang et al., 2013; Rojas et al., 2014; Rana and Musto, 2018). Excess prostaglandins modulate calcium mobilization and cAMP activity, inducing neuronal injury and defects in neuronal plasticity (Hein and O’Banion, 2009; Figueiredo-Pereira et al., 2015; Kang et al., 2017). For example, during febrile seizures, inflammation in the hypothalamic neurons modulate systemic inflammatory response by recruiting prostaglandins from the system (Berg et al., 1998; Zetterström et al., 1998). This enhances EP1/EP2 receptor trafficking, stimulates COX-2 production, and increase prostaglandins within the brain, thereby reducing the threshold for seizures (Gatti et al., 2002). So far, multiple clinical trials of prostaglandin inhibitors for controlling febrile seizures have been largely contradictory: for e.g., patients treated with aspirin therapy had fewer seizures on day two of monitoring, whereas randomized placebo-controlled ibuprofen treatment, in children with febrile seizures, failed to prevent spontaneous recurrent seizures (SRS) (Godfred et al., 2013). An overproduction of prostaglandins and cytokines, along with the recruitment of other disease-causing molecules (such as platelet activating factors, MMPs and TLRs) trigger cellular damage, decrease long-term potentiation, elongate dendritic spines, increase production of forkhead transcription factor 3, modulate voltage-dependent ion channels, and impair BBB leukocyte-endothelium interactions (causing a leaky BBB) (Anderson and Delgado, 2008; Vezzani et al., 2012, 2015; Rana and Musto, 2018). Enhanced production of these molecules and the events they trigger lowers the seizure threshold post-injury and increases the brain’s susceptibility to PTE.

## Immune Response After TBI

Immune cells play important roles in regulating normal functions of the brain, such as neurogenesis, cognition, aging, translation, formation of neural circuits, and stress responses. When this system stops functioning well, disease manifests. Therefore, it is essential to understand the functions of the immune system, to be able to evaluate its role as a repair mechanic that can be optimized, or a disease promoter that should be suppressed. The local inflammation surrounding an injured tissue is pivotal for its recovery. Although sometimes inflammation runs out of control, suppressing it may impact the normal functions of the system. Several studies report that circulating immune cells are vital for CNS protection and repair (Louveau et al., 2015; Morimoto and Nakajima, 2019; Norris and Kipnis, 2019). Blood macrophages are initially activated at the site of injury, and are generally anti-inflammatory and not proinflammatory in nature, challenging the notion of a strictly proinflammatory role for macrophages, post-injury (Popovich et al., 1996). These macrophages are reparative and alternate between an activated or M2 morphology (Rapalino et al., 1998). Rapalino et al. (1998) reported that animals injected with these macrophages, at the site of injury, recovered their locomotor activity and formed less scar tissue. Studies on a TBI chimeric mouse models of neurological diseases have addressed the need for the recruitment of monocytes/blood macrophages to fight progression of the disease, that follows post-injury. These studies proposed that blood macrophages degrade amyloid-β, elevate IL-10 levels, downregulate TNFα, and boost levels of growth factors, such as IGF-1, in the brain, which attenuates neuropathology (Shechter et al., 2009; Hu et al., 2012; Hsieh et al., 2013; Zyśk et al., 2019). Other studies argued that not only do macrophages have a reparative role, but so do circulating *T*-lymphocytes after injury (especially CD4^+^ lymphocytes) (Rapalino et al., 1998; Shechter et al., 2009). For instance, elevating the levels of myelin-recognizing T cells, after TBI, is protective and supports recovery, enhances neurogenesis, improves cognition and provides better protection and the ability to cope with stressful conditions (Mckee and Lukens, 2016; Krämer et al., 2019). It is notable that these protective T cells are different from those that cause autoimmune diseases, in terms of their antigen affinity and regulation. These studies validate an indirect role of T cells in maintaining brain homeostasis by regulating hippocampal neurogenesis, maintaining brain plasticity, enhancing cognition, and controlling the stress response.

Immune cells such as microglia (inflammatory microglia) initiate debris disposal after injury, whereas anti-inflammatory microglia initiate healing in response to sterile inflammation. During severe injury, if microglia cannot clear the debris, macrophages from the blood (or healing macrophages) enter the brain, and terminate the microglial response by releasing high amounts of IL-10. Resident microglia and blood macrophages have different functions in protecting the brain from neuroinflammation and behave differently, in a time-dependent manner. The infiltrating blood macrophages support cell survival and renewal after injury, whereas their depletion causes loss of cells (Shechter et al., 2009). Importantly, immune system activation does not always exacerbate the injury response and cause chronic inflammation. If the activated microglia can return to normal, then inflammation resolves itself; if not, however, they can trigger a systemic immune response. Therefore, it is crucial to understand whether the nature of the inflammation is local or systemic when employing anti-inflammatory therapies. In contrast to systemic inflammation, suppressing local inflammation may prove to be the more beneficial option.

The inflammation conundrum in neurodegenerative diseases occurs in the backdrop of ineffective recruitment or a dysfunctional immune system; it varies with model, strain, sex, region of the brain affected, severity and time of insult, age, etc. After an initial infection, the number of T cells remains steady for a long period of time and then declines; and when their number crosses a critical threshold, disease is manifested. The drop of T lymphocytes over time, as reported by Ho et al. (1995), is a very dynamic process, since immediately after infection T cells furiously regenerate themselves. The inflammatory response then kicks in to regenerate more T cells to fight the infection. When this process is exhausted, the disease is evinced (Ho et al., 1995). Researchers recently discovered that the brain is not as immune privileged as previously thought. In fact, CD4 T cells are present around leptomeninges, blood vessels and glia limitans, where they secrete immune signals into the CSF that bathes the brain; and these cells populate the brain meninges right around the time when all of the synaptic remodeling events are taking place, thereby exacerbating inflammation (Rauch, 2004; Koronyo et al., 2015; Pasciuto et al., 2020). In addition to this neuroinflammatory component, PTE also has a peripheral immune element, as the periphery too gets inflamed by the TBI-activated innate and adaptive responses. After traumatic injury, studies have reported a significant activation of immune cells, such as B cells, CD3+, CD4+, and CD8+ T cells, Tregs, and γδ-T cells in spleen as detected through flow cytometry. Evidence of innate and adaptive responses to injury were also observed in other tissues, such as the GI tract and liver (Tobin et al., 2014; Bai et al., 2017). For instance, PCR-arrays tracking cytokine expression showed increase in mRNA for chemokines, such as MCP-1, in the liver and gut, and proliferation of γδ-T cells (Tobin et al., 2014). Researchers are now beginning to realize that cytokines, for the most part, are not made by the neurons and astrocytes but by immune cells and microglia, which populate the entire body including the developing brain, and communicate with resident macrophages to promote tissue remodeling and cleanup (Röszer, 2015; Sridharan et al., 2015; Wynn and Vannella, 2016; Kumar, 2019). Interestingly, in a recent study on maternal immune activation, it was observed that maternal immune activation (from infection or autoimmune predisposition) induces T-cells to release IL-6 and IL-17. IL-17 can cross the placenta and cause cortical malformation and behavioral abnormalities in the baby (Choi et al., 2016; Wong and Hoeffer, 2018). Together, these studies conclusively support a crucial role for our immune system in health, and in the maintenance of inflammation after TBI.

## Contribution of Astrocytes and Microglia to PTE

### Role of Microglia in TBI/PTE

Microglia are the resident immune cells that play an important role in immune surveillance of the CNS. Based on their morphology and activation, microglia have various subpopulation forms in the CNS. These subtypes include M0, M2a, M2b, M2c, M2d, and M1 (Franco and Fernandez-Suarez, 2015). M2 microglia have anti-inflammatory properties and play a significant role in maintenance of CNS homeostasis and plasticity, synaptic pruning, removal of pathogens through phagocytosis, neural development, regulating neurotransmitter release, neurogenesis, release of neurotrophic factors and tissue/synaptic remodeling. Microglia are acquisitively sensitive to changes in their local microenvironment. They dramatically change their phenotype and upregulate number of diverse cell-surface antigens. These microglia are typically referred to as M1 microglia. M1 microglia are in the hyper-activated state and can be amoeboid or rod-shaped. They promote immune cell recruitment into the CNS (such as Th1 and Th17), where they release proinflammatory cytokines, chemokines such as CCL2 and CCL20, and monocyte chemoattractant protein-1 and eotaxin. Under such circumstances, microglia stimulate iNOS production, trigger generation and release of ROS/RNS, activate the complementary proteins, and increase COX-2 production to produce prostaglandins (Streit et al., 2004; Dheen et al., 2007; Franco and Fernandez-Suarez, 2015).

**FIGURE 5:**
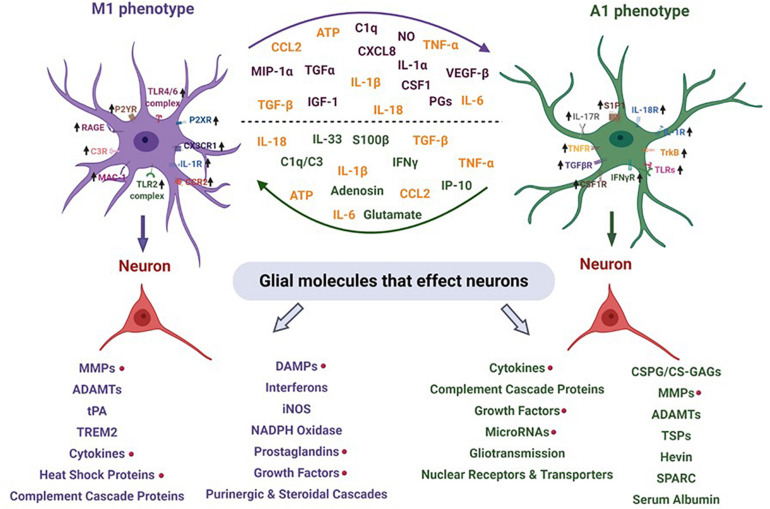
Crosstalk between reactive microglia, reactive astrocytes and neurons during neuroinflammation. During inflammation, microglia and astrocytes achieve M1 and A1 like morphology. M1-like microglia, also known as reactive microglia, secrete multiple factors that modulate astrocytic functions by binding onto their receptors. Likewise, astrocytes too secrete molecules that bind onto microglia and modulate their activities. Molecules in purple are released by microglia and induce A1 phenotype in astrocytes and/or modulate astrocytic activity. Molecules in green are released by astrocytes and modulate microglia activity and/or forms reactive microglia. Molecules in orange are released by both astrocytes and microglia, co-regulating each other’s activities. Molecules released by these cells also effect neurons (in red). Beneath neurons are molecules (in purple) that microglia secrete which cause neuronal damage. In green are molecules secreted by astrocytes that cause neurodegeneration. Red dots next to purple and green text indicate molecules released by neurons that alter microglia and astrocytic activities. Some of the receptors on M1 microglia such as TLR4/6 complex, RAGE, CX3CR1, P2YR and P2XR cause microglia activation due to neural injury-derived DAMPs.

During TBI, M1 microglia express several receptors on its surface as a result of either neural injury-derived damage-associated molecular patterns (DAMPs) or due to astrocytic secretions, that bind onto these receptors. Many of these receptors are a family of pathogen recognition receptors, such as TLRs, that recruit adapter proteins and initiate complex cascade of signaling events which regulate transcriptional events and inflammation. In response to DAMPs, and factors released by damaged neurons, astrocytes and immune cells, microglia drastically changes its morphology, proliferate, move along chemotactic gradience, express surface molecules for signaling, carry out cytotoxic attack on neurons and increase increase proinflammatory secretions. Activation of proinflammatory receptors and their downstream products, when released, either causes neural injury or modulate astrocytic activity, causing an A1 phenotype (Sharma and Naidu, 2016; Clark et al., 2019; Wofford et al., 2019). For instance, primary astrocyte-activating signals released by microglia include IL-1, TNFα, and C1q (Liddelow and Barres, 2017; Clark et al., 2019). These cytokines and complement proteins cause structural and functional changes in astrocytes. Reactive astrocytes disassemble synaptic connections between neurons and release neurotoxins that degenerates mature neurons and oligodendrocytes in CNS after TBI (Liddelow et al., 2017). Astrocytes, likewise microglia, also express high levels of proinflammatory receptors, which similarly alter microglial and neuronal activity. The crosstalk between astrocytes, microglia, and neurons causes degradation of the extracellular matrix (Hevin) and metabolic proteins (ADAMTs), triggers leukocyte mediated inflammatory responses (TREM2, complement proteins), promotes neutrophil chemotaxis (complement proteins), recruits immune cells (purinergic receptors), stimulates cell lysis, enhances production of miRNAs, disrupts lipid homeostasis and cell membranes (nuclear receptors), and impairs synaptogenesis (SPARC) (Figure 5). This cross-coupling between neuroglia induce changes in glial physiology causing long-term neurodegenerative changes after TBI, promoting epileptogenesis resulting in PTE (Smith, 2013; Izzy et al., 2019). Increased inflammasome binding onto TLRs, during the first few days post-injury is one of the major drivers of neuroinflammation that triggers epileptogenesis (O’Brien et al., 2020). In addition to the above events, microglial pruning of synapses is increased very early in the disease progression; as a result, the loss of synaptic density due to an increase in the phagocytic capacity of microglia could perhaps be an important factor that promotes epileptogenesis after TBI (Andoh et al., 2019).

The signals and response modifiers in microglial activation can be triggered and modified by several factors based on the cellular origin, chemical structure and signaling. These include structures of infectious agents, immunoglobulins/immune complexes, complement system, cytokines, neurotrophic factors, proteins and peptides, and neurotransmission related compounds and ions such as ATP, purines and glutamate. Many of these molecules, and associated signaling events, are also released after TBI, which perhaps play a crucial role in the pathogenesis of PTE (Wofford et al., 2019). Signals emitted by neighboring resident cells or by immune cells from the periphery shape profiles of induced genes and functions in microglia. Studies have shown that activation of certain types of cytokines such as IFNγ, IL-1β, and TNFα after TBI, drives proinflammatory microglial response with increased expression of IL-12, supporting the role of immune cell (such as Th1) mediated reactions in regulating M1 state of microglia. These signals, in turn, influence the cocktail of chemoattractive factors to organize for a change in the composition of infiltrates to instruct the engagement of neutrophils, monocytes and distinct T-cell subpopulations (Hanisch and Kettenmann, 2007). Indeed, there are multiple reports of the phenotypic shifts in macrophages and microglia *in vitro* and *in vivo*, and cytokines produced by T-cell subtypes such as IFNγ are primarily known for this change. These series of events can orchestrate inflammatory reactions in response to traumatic insult (Hanisch and Kettenmann, 2007). Furthermore, time lapse images of microglia, after TBI, have revealed immediate microglial response to the focal injury. They undergo a rapid phenotypic change and form a bulbous body that extend towards the ablation site forming a spherical containment releasing ‘on’ signals (Davalos et al., 2005). On signals are inducible factors and includes a range of chemokines, but also neurotransmitters such as purines and glutamate. A prominent feature of reactive microglia is the high expression of receptors for purines and their wide range of responses to receptor activation, which have been reported in numerous experimental models TBI (Davalos et al., 2005; Jackson et al., 2016; Frenguelli, 2019). For instance, single focal injection of ATP in mice induced a localized response of activated microglia with higher P2Y6 and P2Y12 receptor expression, may support the role of purine receptors in TBI induced epileptogenesis (Davalos et al., 2005; Kumaria et al., 2008; Jackson et al., 2016).

The exciting development in microglia research in terms of origin and progenitors of microglia, their population and stability or turnover under normal and diseased conditions, their contribution to the maturation and support of neuronal development and glial functions, their protective and harmful actions in diseases and the options of therapeutic interventions by silencing or enhancing functions, will help to answer several key questions and help in understanding their role more clearly in health and disease.

### Astrocytes and Their Role in TBI/PTE

Astrocytes, first identified by Virchow in 1846 as a glue filling the interstitial fluid, are star-shaped cells in the CNS that play an important role in maintaining brain homeostasis. Astrocytes use their “astrocytic end-feet” to support the metabolic demands of neurons by supplying nutrients from the blood vessels (Cui et al., 2012). They make ‘tripartite synapses’ with pre- and post-synaptic neurons, to integrate synaptic function by means of neurotransmitters and gliotransmitter release (Cui et al., 2012). Neuroactive molecules of astrocytes, such as_*D*_-serine, GABA, and adenosine triphosphate (ATP), regulate neuronal functions such as synaptic activity by inducing long-term depression and long-term potentiation, mediate tonic inhibition through Best1 ion channels, inhibit proinflammatory molecules (such as TNFα), assist GABA transporters in a calcium-independent manner and regulate sleep homeostasis, synaptic plasticity, and memory formation (Panatier et al., 2006; Haskó et al., 2008; Lee et al., 2010; Yoon et al., 2011; Fossat et al., 2012).

Astrocytes regulate neuronal functions under normal physiological conditions, but under pathological conditions, astrocytes phenotypically change in response to their microenvironment and become reactive during inflammation (Cui et al., 2012; Pekny and Pekna, 2016). After TBI, reactive astrocytes undergo morphological changes, that corresponds to changes in their functional and molecular properties. These alterations include dysfunctional potassium and glutamate buffering, modulation of aquaporins and adenosine activity, disturbances in gap junctions, disruption of glutamate-glutamine cycle, impairment of cysteine-glutamate antiporter system and mutations in potassium channel genes (Lewerenz et al., 2013; Burda et al., 2016; Zhou et al., 2020). Series of these events over time, results in the accumulation of neurotoxic molecules in the brain and cause BBB disruption. The damage to the BBB promotes extravasation of serum albumin into the brain (Puttachary et al., 2016). Serum albumin in the brain binds to TGFβ receptors on astrocytes, which phosphorylates Alk5 mediated SMAD2/3 complex and p-38 MAPK. This causes SMAD2/3 translocation into the nucleus activating transcription of proinflammatory genes promoting TGFβ and IL-6 production (Milikovsky et al., 2017). In numerous studies on rodent models of TBI, it has been reported that extravasation of serum albumin cause impairment of potassium buffering and glutamate reuptake by downregulating Kir4.1 potassium channels and glutamate transporters (Ranaivo et al., 2012; Weissberg et al., 2015; Zhou et al., 2020). This elevates extracellular K^+^ and glutamate concentration, and cause hyperexcitability (Puttachary et al., 2016; Steinhäuser et al., 2016). Infiltration of peripheral immune cells (such as T cells and monocytes) after BBB breakdown signals the release of complementary proteins. Up-regulation in complementary proteins promote leukocyte chemotaxis and migration at the lesion site (Cho, 2019). Many complement cascade genes are profoundly upregulated in the reactive astrocytes and neurons after TBI. They play an important roles in activating numerous pathological pathways involved in synaptic loss, increased synaptic pruning, impairment of neuromelanin clearance, increased stress in endoplasmic reticulum, decreased phagocytosis by dendritic cells, modulation in neurite outgrowth and regulating control of growth factors (Daglas and Adlard, 2018; Hammad et al., 2018; Cho, 2019). In response to proinflammatory insult, reactive astrocytes also produce an unknown factor called protein-X, which triggers the production and shedding of the complement components by neurons (Shi et al., 2010). Excessive tagging of neurons by activated complement proteins and their recognition by complement receptors or reactive microglia results in phagocytosis and removal of synapses, and eventually neuronal death.

The combination of aforementioned damaged signals and their relative concentrations most likely determine the type of astrogliosis experienced by astrocytes in different regions surrounding the initial insult zone. On a cellular level, insult to the brain such as TBI results in hypertrophy of astroglial processes and significant increase in astrocytic cytoskeleton (Sofroniew and Vinters, 2010; Burda et al., 2016; Steinhäuser et al., 2016; Chen et al., 2020). Brain damage very rapidly turns most of the astroglial cells into GFAP expressing reactive astrocytes. Both GFAP and vimentin are critically important for the development of reactive astrocytes. Severe stress in astroglia energetics leads to subsequent loss of ion homeostasis that triggers enormous amounts of glutamate in ECS. The astroglial involvement in controlling brain glutamate concentration is double edged. Upon severe injury, astrocytes may turn from being the sink for glutamate to being the main source of the latter. Astrocytes can release glutamate by several mechanisms which are triggered in PTE. First, the reversal of glutamate transporters can be caused by ATP depletion accompanied with an increase in intracellular Na^+^ concentration and cell depolarization. Second, elevation of cytoplasmic Ca^2+^ concentration in astrocytes, that follows traumatic injury, may trigger the release of glutamate stored in vesicles. Third, acidosis and lowering extracellular Ca^2+^ concentration may open glutamate-permeable hemichannels. Fourth, ATP released in higher concentrations by dying and disintegrating neurons can open astrocytic P2X_7_ purinoceptors which allows glutamate release. Fifth, brain oedema post-TBI can activate volume-sensitive channels which too allows the passage of glutamate.

Excess glutamate in the extracellular space disrupts the cysteine/glutamate antiporter system (CGS), a key anti-oxidant system in astrocytes that imports oxidized cysteine into the cell in exchange for glutamate. CGS regulates movement of amino acids in to and out of the cell, depending on the cellular requirements, and regulates the immune system, resistance against anti-cancer drugs, protection against carcinogenesis, cellular redox homeostasis, and modulates memory and behavior. In astrocytes, the intracellular concentration of cysteine (in its reduced form) is generally lower than glutamate (Lewerenz et al., 2013). Cysteine is an important substrate for the production of glutathione; and, inside the cell, oxidized cystine is reduced to form glutathione through the help of enzyme thioredoxin reductase 1 (Mandal et al., 2010; Lewerenz et al., 2013). Post-TBI, dysfunctional CGS upsets the balance between anti-oxidant and oxidants, causing oxidative stress as a result of glutamate excitotoxicity (Koza and Linseman, 2019). During inhibition of CGS, glutathione levels decline. Once glutathione depletion reaches a critical level, ROS production increases. This does not cause cell death immediately but instead facilitates the activation of signaling pathways and ultimately culminates in cell death. Therefore, neuroprotective compounds that generally are not beneficial during chronic stages of the disease can have favorable outcomes when administered at early time-points (when ROS concentration is gradually increasing, post injury) (Maher and Schubert, 2000; Lewerenz et al., 2013). Modulation in CGS levels have been reported in many neurodegenerative conditions. The increase in CGS in these conditions could primarily be due to an increased glutamate accumulation and release (Chung et al., 2005; Pampliega et al., 2011). This rise in extracellular glutamate is a result of downregulation of the excitatory amino acid transporter (EAAT) that balances CGS-mediated glutamate release. Therefore, specifically targeting glutamate by inhibiting CGS, can be an alternate approach for treating TBI related disorders as it aims to balance glutamate release into the ECS with glutamate uptake by EAATs. Drugs that protect from glutamate excitotoxicity act mainly through these mechanisms and inhibit excitotoxic effects of CGS by increasing glutathione synthesis modulating glutamate release (Lewerenz et al., 2013).

After TBI, initiation of secondary insult mechanisms can trigger epileptogenesis. If primary mechanisms are not controlled on time, they can cause long-term cellular and molecular alterations in astrocytes, leading to serious neurological consequences over time. For instance, astrocytic dysfunction can result in disruption of homeostatic regulation of brain volume and water content levels, causing edema. This can result in increased intracranial pressure, changes in extracellular osmotic pressure and compression damage to neural tissues (Dearden, 1992; Jha et al., 2019). After TBI, astrocytes are unable to remove excess water due to damage to their water channels, called aquaporins. Aquaporins have been widely studied as drivers of pathogenesis in epilepsy and other neurodegenerative conditions. Mutations in aquaporin 4 disrupt fluid osmolarity and potassium homeostasis (Heuser et al., 2010; Binder et al., 2012; Nagelhus and Ottersen, 2013). Although the role of gap junction dysregulation in epilepsy is still controversial, some studies have demonstrated an anti-epileptic role of gap junctions during astrocytic coupling. According to the spatial buffering concept, astrocytes pass excess K^+^ ions between their networks, reducing K^+^ concentration in the ECS. Dysfunctions in gap junction proteins, such as connexins, have been reported to increase cellular hyperexcitability and cause seizures. For instance, studies on Cx30^–/–^ mice reported increased neuronal depolarization and lower seizure threshold with disturbances in potassium and glutamate clearance in astrocytes, causing astrocytic swelling (Wallraff et al., 2006; Steinhäuser et al., 2016). Damage to aquaporins and rapid swelling of astrocytes after injury is accompanied by a significant increase in astroglial surface area. Astroglial swelling can trigger numerous secondary effects that can exacerbate the brain damage. In particular, swelling of perivascular astrocytes and astrocyte endfeet may compress brain vessels and limit circulation. Swelling of astrocytes can result in the opening of volume regulated ion channels permeable to glutamate and other excitatory amino acids exacerbating excitotoxic cell death (Sun et al., 2003; Tran et al., 2010). Therefore, the functional and molecular changes in astrocytes, after TBI, promote epileptogenesis suggesting their role in the development of PTE.

## Long-Term Consequences of TBI/PTE

The possibility of developing PTE, after post-traumatic seizures, is generally higher and so increases the risk of long-term consequences of TBI. These consequences depend on the severity of injury and the region of the brain affected. For instance, the odds of developing long-term implications diminish if the injury is a mild or moderate closed-head one, in contrast to a severe closed-head injury (Naalt et al., 1999; McCullagh and Feinstein, 2003). The closed-head TBI causes bleeding or intracranial hematoma which raises the risk of lasting impact on the brain. Recently, a 30 years followup study conducted in Sweden on patients with TBI, reported that all survivors of TBI, whether moderate or severe, developed dementia within 30 years of injury (Himanen et al., 2006). Moreover, studies on Vietnam War veterans, over the span of 40 years, revealed that treating with anti-convulsants during the acute phase of injury controlled severity and frequency of early seizures, whereas later treatments with anti-convulsants did not prevent the onset of PTE (Raymont et al., 2011). Over 40% of troops that suffer TBI develop PTE in their lifetime, with lasting effects including confusion, cognitive deficits, depression, and anxiety disorders. Long-term follow-up studies on veterans also revealed that about 18% of veterans experienced their first seizure after 15 years of injury (most had seizures after 1–5 years) and about two-thirds are on life-long medications (Raymont et al., 2010, 2011). Interestingly, having a family history of epilepsy or a genetic predisposition adds to the risk of developing PTE after brain injury. The genes involved mainly control plasticity, modulate levels of neurotransmitters, control ion channels, and regulate immune functions (Swartz et al., 2006; Raymont et al., 2010).

After TBI, the incidence of PTE increases and some patients are notoriously difficult to treat due to challenges in long-term follow up and therapeutics (Garga and Lowenstein, 2006; Schmidt et al., 2014; Szaflarski et al., 2014). Video-EEG monitoring and MRI studies on patients with PTE reported that approximately one quarter of the patients develop mesial temporal sclerosis and predicted the development of neocortical lesions on other half of the patients, at some stage in life; whereas, the vast majority of the cases develop focal epilepsy (Gupta et al., 2014). Swartz et al. reported that, of 200 consecutive temporal lobectomies performed on TBI survivors, 21 cases were of PTE, and about 50% of these cases had hippocampal sclerosis characterized by neuronal loss primarily in the hilar region of DG (Swartz et al., 2006). Moreover, a CEEG and PET scanning on 16 TBI patients revealed that ∼28% of these patients had non-convulsive seizures (NCS) over 7 days after injury and one had R temporal NCS during PET while comatosed (Vespa et al., 2010). Further, the same study reported the patients who had seizures several days after injury developed hippocampal atrophy, ipsilateral to the seizure, which was possibly why some develop PTE later in life (Vespa et al., 2010). High-resolution analysis of the brain (through diffusion tensor imaging of the perforant path) revealed that the white matter tracts that are either afferent and efferent to the hippocampus are particularly sensitive to shearing and stretching forces (Wang et al., 2008). This indicates that, at least in some cases, the mechanism of epileptogenesis results from a deafferentation or disconnection of the hippocampus from the long-term synaptic connections which develops over time.

Repeated TBI can alter neural circuits and lead to long-term degenerative changes in the brain and periphery. For instance, chronic traumatic encephalopathy (CTE; a neurological deterioration due to accumulation of hyperphosphorylated tau) causes release of TDP43 (transactive response DNA binding protein), which forms neurofibrillary tangles and increases oxidative stress. TDP43 is produced in high amounts, which affects the anti-oxidant enzyme SOD-1 and causes protein misfolding, damaging the BBB (Pokrishevsky et al., 2012). The breakdown of the BBB may persist for many months or years, gradually causing damage over time. The BBB disruption results in local inflammation which ultimately resolves into epileptogenesis (Tomkins et al., 2011; Vezzani et al., 2012). In the periphery, the cardiac complications of PTE cause morbidity and mortality due to enhanced cardiac contractility, high blood pressure, and production of myocardial ROS. The increased cardiac contractility results in sympathetic storm that causes arrhythmias, high blood pressure, reduced heart rate variability, and the manifestation of congenital heart problems. It also raises plasma catecholamine production, further damaging the myocardium (Shanlin et al., 1988). Elevated catecholamine enhances oxidative load in myocardial tissues, disrupting the balance between oxidants and anti-oxidants. This diminishes NO bioavailability in the heart, affecting general circulation and regulation of blood pressure (Larson et al., 2012).

Generally, after penetrating or severe closed TBI, altered homeostatic mechanisms generate the first seizure, usually a generalized seizure with focal onset, and a late seizure that is a partial complex seizure. A better understanding of the molecular mechanisms that cause these seizures and epilepsies is imperative for development of better drugs and treatments. Moreover, greater understanding of the brain’s immune system is also necessary to identify the causal mechanisms of long-term PTE-related consequences.

## Therapeutic Intervention and Management

Management of brain injury focuses primarily on preventing signs of secondary injury. Currently, no therapies are available for permanently treating TBI-related injuries, although more than 20 drugs are available to treat epilepsy (however one-third of epilepsy patients are refractory to these drugs) (Dalic and Cook, 2016; Hogan, 2018). Moreover, over 40 failed drugs have been tested in the clinical trials against epilepsy in the past decade, most of which were ion channel targets. The failure of these compounds to treat PTE could perhaps be due to the complexity of PTE and the new unknown mechanisms that regulate epileptogenesis after TBI (Temkin, 2001; Varvel et al., 2014). Therefore, it is important to investigate novel non-neuronal targets/mechanisms other than ion channels, such as enzymes, glial cells, neurovascular components, oxidative stress molecules, and nuclear proteins (Table 2).

**TABLE 2:** Novel therapeutic interventions that may have potential to impact the outcome of TBI-induced epileptogenesis.

Treatment	Model	Specie, age, strain	Dosage regimen	Targets/suggested mechanism of action	Region/Tissue analyzed	Outcome/effects	References
**ISO1**	Lateral Fluid Percussion Injury	Mice, 8 weeks, C57BL/6J	10 mg/kg (i.p.) (single dose), 30 min post-injury	• Macrophage migration inhibitory factor (MIF) antagonist • Inhibits MIF binding to CD74 and prevents its cleavage and activation • Inhibits TNFα and reduces gliosis	Parietal CTX; Brain leukocytes; Intestinal lymphocytes	• Decreased astrocyte activation and B cell brain infiltration • Elevation of splenic B cells • Inhibition of γδT cells’ increase in gut	Newell-Rogers et al., 2020
**Baicalein**	FeCl_3_-induced injury	Male, 18–22 g, C57BL/6J	50 and 100 mg/kg (i.p.) (single dose), 30 min prior to injury	• Positive allosteric modulator of GABA_*A*_ receptor • Inhibitor of CYP2C9 and prolyl endopeptidase • Inhibits lipoxygenases	Somatosensory cortex; Hippocampal HT22 cells	• Reduced number and duration of seizures • Reduction in FeCl_3_-induced PTS • Inhibition of 12/15-LOX-mediated lipid peroxidation by • antagonizing ferroptosis • Neuroprotection against FAC-induced HT22 cell damage	Li et al., 2019
**Ceftriaxone**	Lateral Fluid Percussion Injury	Rat, 8–9 weeks, Long-Evans	200 mg/kg (i.p.) for 7 days (once daily), 30 min post-TBI	• Third-generation cephalosporin antibiotic, also anti-microbial in nature • Inhibits mucopeptide synthesis in bacterial cell wall by binding to carboxypeptidases, endopeptidases, and transpeptidases	Neocortex	• Reduced seizures • Restoration of GLT-1 expression and reduced gliosis in lesioned cortex • Attenuation of PTS	Goodrich et al., 2013
**Creatinine**	Fluid Percussion Injury with PTZ	Rat, 250–300 g, Wistar	300 mg/kg (oral) for 3–7 days (once daily), 30 min post-TBI	• Neuroprotective, anti-inflammatory and cardioprotective actions • Inhibits JAK/STAT1 signal transmission by inhibiting interaction of IFNγ receptors with JAK2	Parietal CTX	• No change in susceptibility to seizures • Protection against protein carbonylation and TBARS after neuronal damage • No effect on convulsive parameters	Saraiva et al., 2012
**Ketogenic diet**	Fluid Percussion Injury with Flurothyl-induced seizures	Rat, 8 weeks, Sprague Dawley	Bio-Serv F3666 diet for 9 weeks, started 3 weeks prior to TBI	• High fat low carbohydrate diet, effective against drug-resistant epilepsy • Generates ketone bodies- which has anti-convulsive properties	Hippocampus	• Primarily anticonvulsive but not antiepileptogenic • Increased latency to myoclonic jerks at 9 weeks • Protection against hippocampal lesion volume and cell loss • Reduced gliosis and MFS	Schwartzkroin et al., 2010
				• Increases GABA, NPY, adenosine and reduces glutamate			
**Apocynin**	Lateral Fluid Percussion Injury	Mice, 28–32 g, Swiss	0.05, 0.5, and 5 mg/kg (subcut) (single dose), 30 min and 24 h post-TBI	• NADPH-oxidase inhibitor • Reduces pro-inflammatory cytokine production, neutrophil infiltration, ICAM-1 and P-selectin expression, PAR and nitrotyrosine formation, and MAPK activation	Cortex	• Attenuation of IL-1β, TNFα, NO metabolites and water content levels • Reduced oxidative damage (protein carbonyl, lipoperoxidation) • Reduced cortical lesion volume • Reduced secondary brain damage and improved cognition	Ferreira et al., 2013
**Minozac**	Closed Head Injury with Electroconvulsive Shock	Mice, 20–25 g, CD-1	5 mg/kg (i.p.) (two doses), 3 and 6 h post-TBI	• Selective inhibitor of proinflammatory cytokine by activated glia • More potency towards IL-1β, TNFα, and IL-6	Hippocampus	• Reduced seizure • susceptibility and neuronal injury by suppressing cytokine elevation • Diminished astrocyte activation and metallothionein expression • Improved neurobehavioral task performance	Chrzaszcz et al., 2010
**Monophosphoryl Lipid A (MPL) and Pam3Cys**	Controlled Cortical Impact with electrical kindling	Rat, 9 weeks, Wistar	1 μg/1 μl/rat MPL and Pam3Cys (intracerebroventricular injection) in lateral ventricle, 5 days prior to TBI	• Toll-like receptor agonists and potent stimulator of T-cells and antibody responses • Affects adaptive immune responses via specific interactions with B cells • Activators of monocytes and macrophages	Parietal cortex	• Reduced acceleration of epileptogenesis caused by trauma • Reduction in TNFα levels • No change in the speed of kindling and duration of kindled seizure parameters • Prevented decrease in seizure threshold	Hesam et al., 2018
**Pyrroloquinoline Quinone (PQQ)**	Controlled Cortical Impact	Rat, 8–9 weeks, Sprague Dawley	5, 7, and 10 mg/kg (i.p.) (single dose for 3 days) prior to TBI	• Superoxide scavenger and prevents oxidative changes • Inhibits glutamate decarboxylase and protects against NMDAR mediated neurotoxicity • Increases nerve growth factor synthesis	Cortex; Hippocampus	• Reduced oxidative stress induced neuronal death • Diminished cortical lesion volume • Reduced destruction, disordered arrangement and abnormal nuclear morphology in CA2 • Improved spatial memory and learning performance • Enhanced β-1,4-GaIT-I and -V expression and 4-GlcNAc in microglia and neurons	Zhang et al., 2012
**Rapamycin**	Controlled Cortical Impact	Mice, 8 weeks, CD-1	6 mg/kg (i.p.) (single dose for 4 weeks), 1 h post-TBI	• Specifically inhibits mTOR by forming immunosuppresive complex with FKBP-12 • Inhibits T-cell activation and proliferation that occurs in response to proinflammatory cytokine stimulation	Neocortex; Hippocampus	• Reversed hyperactivation of mTORC1 pathway • Decreased neuronal • degeneration and mossy fiber sprouting • Reduced seizure frequency and rate of developing PTE	Guo et al., 2013
**SR141716A/Rimonabant**	Lateral Fluid Percussion Injury	Rat, P21–22, Wistar	1 and 10 mg/kg (i.p.) (single dose), 2 and 20 min post-TBI	• Selective CB1 antagonist and a dual inhibitor of ACAT • Alters cell cycle distribution and produces G2/M cell cycle arrest • Modulates RANTES and MCP-1 levels • Attenuates and controls neutrophils, monocytes and PDGF levels	Cortex	• Reduced post-traumatic hyperexcitability • Attenuation in long term increase in seizure susceptibility • Increased seizure latency and reduction in cumulative duration of seizures	Echegoyen et al., 2009
**Trametinib**	Controlled Cortical Impact	Mice, C57BL.6J	1 mg/kg (oral) (single dose for 7 days), 2 h post-TBI	• Highly specific and potent MEK1/2 inhibitor • Inhibits cell proliferation, activates autophagy and induces apoptosis	Cortex; Primary microglia culture	• Rescued oligodendrocytes and decreased infiltrating microglial density • Reduced microglial activation and proinflammatory cytokines • Inhibition of microglial MEK/ERK signaling cascade activation • Improved cognitive functions	Huang et al., 2020
**DHEAS (Dehydroepian- drosterone Sulfate)**	Weight Drop	Mice, 30–40 g, ICR	20 mg/kg (subcut) (once a week), 7 days post-TBI	• Androgen receptor antagonist and estrogen receptor agonist	Frontal cortex; Hippocampus	• Improved long-term cognitive and behavioral deficits	Milman et al., 2007
**Atipamezole**	Lateral Fluid Percussion Injury	Rat, 12 weeks, Sprague-Dawley	1 mg/kg (i.p.) followed by 100 μg/kg/h (subcut) (for 9 weeks), 30 min post-TBI	• α2-adrenergic receptor antagonist • Reverses analgesia by blocking norepinephrine feedback inhibition on nociceptors	Cortex; Hippocampus	• Reduced seizure susceptibility • Improved cognitive performance	Nissinen et al., 2017
**Gabapentin**	Undercut cortex model	Rat, P30, Sprague-Dawley	100 mg/kg (subcut) (thrice a day for 2 days) and 120 mg/kg/d (subcut) (13–15 days), 1 h post-TBI	• Inhibits L-type calcium channel and thrombospondin-induced excitatory synapses formation • Acts on adenosine receptors and voltage-gated potassium channels	Cortex; Brain slices	• Reduced posttraumatic hyperexcitability • Decreased incidence of evoked epileptiform discharges in cortical slices • Reduced expression of neurofilament and GFAP immunoreactivity • Reduced frequency of spontaneous and miniature EPSCs on layer V pyramidal neurons	Li et al., 2012
**Sodium selenate**	Lateral Fluid Percussion Injury	Rat, Adult Long-Evans	1 mg/kg (subcut) (for 12 weeks), after TBI	• Acts as an antioxidant via actions of selenoproteins for protection against oxidative stress • Acts as a catalyst for the production of thyroid hormone • Activates PP2A and decreases p-tau	Cortex; Hippocampus; Amygdala	• Suppressed epileptogenesis and reduced seizure frequency • Upregulation of PP2A and increased PR55 expression • Decreased tau phosphorylation and neurodegeneration	Liu et al., 2016

No existing treatments can prevent the long-lasting neurodegenerative changes in PTE, but targeting free radicals during the acute phase of inflammation might prove to be more effective. For example, increased levels of NADPH oxidase after TBI damages mitochondria and other organelles (Bordt and Polster, 2014; Angeloni et al., 2015; Ma et al., 2017). Pharmacological inhibitors of free radicals such as NOX, peroxides, peroxynitrites/nitrates, hypochlorites, phenols and prostanoid antagonists can modulate free radical production and suppress inflammation (Cheng et al., 2012; Korkina et al., 2016; Ma et al., 2017; Smith et al., 2019). Studies have shown that inhibition or genetic ablation of NADPH oxidase improves outcomes in terms of neurodegeneration, oxidative stress mediated mitochondrial dysfunction, gliosis, and increases neurogenesis (Cheng et al., 2012; Altenhöfer et al., 2015; Hirano et al., 2015; Maqbool et al., 2020). In contrast to reducing pro-oxidant levels, increasing anti-oxidants can be a useful, alternate therapy for preventing long-term changes in the brain post-TBI. Inhibitors of conventional targets, such as COX-2, IFN, and prostaglandin, can also help in combating poor outcomes. Interestingly, the chronic PTE signature is quite similar to the IFN signature, in terms of activation of neurodegenerative mechanisms; and inactivating type I IFN with αIFNAR-infusion therapy can block IFNα/IFNβ signaling, lowers expression of inflammatory mediators, diminishes neurodegeneration, and attenuates inflammation. Moreover, an ICV infusion of αIFNAR significantly improves cognitive deficits, motor functions, upregulates neuroprotective genes, and reduces lesion volume (Barrett et al., 2020).

Inhibiting hippocampal neurogenesis after TBI can be a viable therapeutic option for preventing mesial TLE. In most cases, the process of neurogenesis in the hippocampus after injury benefits the brain and allows for recovery of memory and normal behavior (Parent, 2002; Sun, 2014; Redell et al., 2020). Some of that repair may be imperfect and show synaptic reorganization of neural networks, which can create circuitry that is epileptogenic. These considerations show the need for caution and careful design of strategies that target aberrant neurogenesis and synaptogenesis, while leaving the neurogenesis and synaptogenesis that are important for recovery in place.

In terms of treatment and management, controlling swelling and preventing hypoxia or ischemia can prove to be another effective therapy. If, at a certain point, intracranial pressure (ICP) rises dramatically with an increase in intracranial volume, then the brain swells substantially. Preventing this is important because the degree of ICP directly correlates with cerebral perfusion pressure (CPP), the key pressure required by brain, in terms of delivery of oxygen and other nutrients. As ICP rises, CPP drops, and as with it the delivery of nutrients and oxygen decelerates having deleterious effects on the brain. This can further affect cerebral blood flow, reducing oxygen content in the blood (Bouzat et al., 2013; Kinoshita, 2016). Therefore, it is important to develop techniques that will sustain cerebral blood at an appropriate level—keeping CPP up without increasing ICP—to prevent permanent infarction from TBI (Zauner et al., 2002). Strategies for lowering ICP may include stepwise medical management or surgical options (like placing a ventricular drain for CSF) or employing strategies that would increase the intracranial vault size (like decompressive craniotomy). Medical management therapies can include normothermia, normoglycemia, targeting blood pressure, maintaining oxygen and carbon dioxide saturation levels, eliminating hyponatremia, decreasing cerebral metabolic demand/rate, increasing the mean arterial pressure to enhance CPP, and use of vasopressors such as norepinephrine and dopamine to prevent edema (Vespa et al., 1998; Hutchinson et al., 2002; Sookplung et al., 2011).

Prophylactic use of antiepileptic drugs (AEDs) can reduce the risk of early post-traumatic seizures but not the later ones. PTE perhaps represents the ideal model to study the mechanisms of epileptogenesis and develop therapies for epilepsy. Over the last several years it has become clear that, properly chosen, cases of PTE can successfully be treated with surgical interventions. Approximately 60% of the surgical cases of PTE end up being Engel class I, ∼20% Engel class II; and about 80% had favorable surgical outcomes. Gupta et al. (2014) reported that patients with mesial temporal sclerosis, as an epilepsy syndrome, have 92% class I and II outcomes. The lesional cases, from both frontal and temporal, and non-lesional cases were somewhat less favorable (Gupta et al., 2014). In contrast, the outcome of surgical intervention depended on the seizure-onset localization zone. The surgical intervention is not generally recommended in most cases of PTE, as seizure foci can be difficult to localize due to technical issues, such as craniotomies and breach rhythms. Patients with severe TBI may have undergone craniotomy, which may cause breath rhythms (special EEG rhythms that can be artifacts or misguide diagnosis). Moreover, patients with severe TBI may have diffuse cerebral or axonal injury evident on EEG recording as multiple epileptic foci that can overlap with localization of eloquent brain regions (Hakimian et al., 2012). Therefore, it is important to carefully select surgical candidates, as the patient with severe TBI may be at greater risk of surgical complications due to their structural damage or scar tissue and adhesion formation. For these reasons, other adjunctive treatment options such as stimulation of the vagus nerve, responsive nerve, and anterior nerve should be considered.

Medical treatment for PTE can be similar to that for other epilepsies, with the caveat that unnecessary treatment with AEDs may impair neurorehabilitation. There is no evidence that treatment with AEDs or anti-convulsants will be beneficial in cases of moderate to severe TBI; however, some evidence suggests AEDs could possibly reduce the incidence of post-traumatic seizure if administered during the acute phase of injury. One study conducted by Hernandez (1997) showed that treating patients with phenytoin during the acute phase lowers the incidence of early post-traumatic seizures from 14.2 to 3.6%. Yet continuing AED treatment beyond the acute phase has never been shown to change the prognosis for the ultimate development of epilepsy (Hernandez, 1997). Recently, levetiracetam (LEV) has gained popularity in the treatment of PTE, as LEV does not cause the same side effects and has lower cytotoxic effects than other AEDs. Even though no randomized controlled trials have been done, comparative studies show the efficacy of LEV seems slightly better than standard AEDs, and with fewer side-effects. On the downside, observational studies report that LEV is not effective in reducing PTE risk, so it is generally not recommended beyond 7 days post-injury (Szaflarski et al., 2010). As an alternate to LEV, sodium channel blockers are an ideal option, but some of these blockers do not appear to be effective against PTE. For example, Dilantin blocks early seizures but it is not regarded as an effective anti-epileptogenic agent (Szaflarski et al., 2010). Although not every sodium channel blocker has been tested in PTE models, additional replacement therapies are required to treat PTE. These alternate therapies must rely on things like blocking inflammation, promoting BBB repair, or perhaps promoting the integrity of damaged axonal pathways to prevent persistent brain inflammation. Therefore, the goal of developing anti-epileptogenic compounds should be tied to these strategies to promote integrity, resilience, and recovery of neural structures.

## Concluding Remarks

**FIGURE 6:**
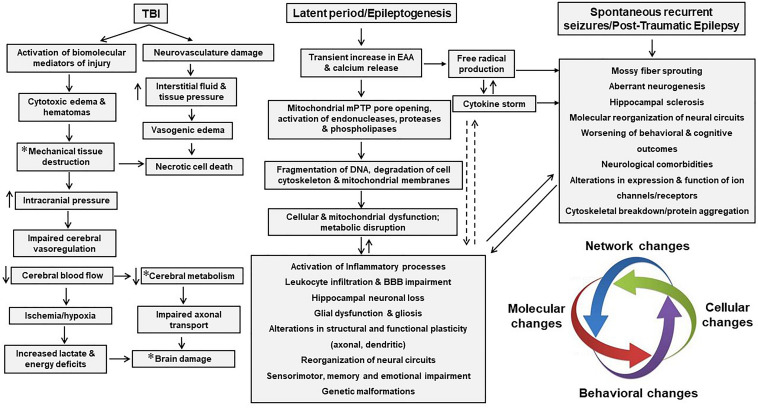
Pathophysiological cascades of events from TBI to PTE. A series of cellular and molecular events overwhelms the brain following TBI. TBI induces neurovascular damage and activates biomolecular mediators of injury. This neurovascular damage leads to vasogenic edema and necrotic cell death, while biomolecular mediators lead to necrotic cell death and brain damage. TBI also induces structural damage that leads to cerebral edema and decreases cerebral blood flow, causing acute neurological damage. In addition, cytokines and other molecules released in the extracellular space trigger free radical production in neurons and glial cells, promotes neuronal excitability, and neuronal death. Alterations in mitochondrial bioenergetics, fragmentation of DNA and structural proteins, and activation of neuroinflammatory pathways cause structural and functional changes in the brain that accompany recurrent post-epileptic seizures. All these events, after TBI, are interwoven and rely on other altered cellular and molecular events which exacerbates post-epileptic seizures. Asterisk represents changes that can also lead to secondary damage either directly or indirectly.

Four key elements—excitotoxicity, neuroinflammation, oxidative stress, and neurodegeneration—are the primary pathognomonic mechanisms responsible for PTE; and it is well known that TBI initiate cycles of neuroinflammatory events that elicit the oxidative stress response tripping a series of events and cycles that exacerbate the acute stage and lead to chronic conditions (Figure 6). The goal of this review is to understand the mechanisms of epileptogenesis after TBI and identify, develop, and validate therapeutic strategies to prevent PTE. In this regard, we can make several key conclusions: (1) the primary source of cellular excitotoxicity after TBI is elevation in extracellular glutamate, increased immune cell infiltration and crosstalk between glial cells and neurons governed mainly through cytokine and chemokine networks; (2) the initial immune response to injury is beneficial and, it works to counterbalance the disequilibrium in the system; (3) Impairment of mitochondria due to an excessive generation of ROS/RNS is a continuous process during epileptogenesis, and is associated with inflammation and neurodegeneration; (4) pro-inflammatory cytokines, and chemokines are the key players released by invading blood cells, microglia, astrocytes, and neurons; (5) over-production of cytokines, lipids, and chemokines over long periods of time triggers cell death; (6) invasion by leukocytes and activated microglia leads to tissue damage at later stages.

Post-traumatic epilepsy is phenotypically heterogenous in humans and it is important to understand this phenotypic heterogeneity to develop antiepileptogenic therapies. Both focal and diffuse mechanisms can result in PTE, and approximately 25–30% of PTE cases are associated with mesial temporal sclerosis. Although surgery is an alternate option, it is generally not recommended, so AEDs remain the first line of treatment. Yet, AEDs are not very effective in treating PTE, but are rather used to treat the symptoms without improving the underlying condition. Patients receiving AEDs often require lifelong AED treatment and some develop severe side effects over time. Furthermore, failure to control epileptic seizures can also lead to increased mortality, reduced quality of life, comorbidity, and depression. In spite of the many AEDs available, a little progress has been made in preventing the onset of new types of epilepsies. Moreover, repurposing anti-seizure drugs to prevent the onset of epilepsy has been entirely unsuccessful up to now.

Understanding epilepsy as a network disorder suggests early phases of epileptogenesis should be targeted before the imbalance spreads to other regions of the brain. This, however, is not as simple as it seems because many of the candidate compounds being investigated have multiple effects and target multiple pathways. These pathways can be different in humans and animal models, which devalues the translational significance of the latter and highlights the importance of designing experiments with the right timing, dosage, and targets, and with appropriate animal models (Smith, 2016; Saletti et al., 2019). Moreover, it is important to define targets with variable injury mechanics and to vary treatments at particular time-points. Ultimately, a clearer understanding of the molecular mechanisms of epilepsy will allow development of truly novel therapeutic targets for PTE.

## Author Contributions

SS reviewed the literature, wrote and edited manuscript, and prepared the tables and figures. GT and JH edited the manuscript. AB conceived the idea, and reviewed and edited manuscript. All the authors contributed to the manuscript revision, read, and approved the submitted version.

## Conflict of Interest

The authors declare that the research was conducted in the absence of any commercial or financial relationships that could be construed as a potential conflict of interest.
